# Comparative analysis of alkyne- and desthiobiotinylated photoaffinity probes for chemotranscriptomic profiling

**DOI:** 10.1039/d6cb00030d

**Published:** 2026-03-04

**Authors:** Daphne A. L. van den Homberg, Georgia Poulladofonou, Aurelia Dekens, Willem A. Velema

**Affiliations:** a Institute for Molecules and Materials, Radboud University, Heyendaalseweg 135 6525 AJ Nijmegen The Netherlands willem.velema@ru.nl

## Abstract

Understanding small molecule–RNA interactions is a crucial part in drug development and fundamental biology. Chemotranscriptomic profiling is emerging as a powerful platform to interrogate interactions of small molecules with entire transcriptomes. This technique relies on photoaffinity probes that covalently capture small molecule RNA interactions. Most photoaffinity probes bear an alkyne handle that requires additional inefficient functionalization and purification steps after RNA capture. We sought to improve the workflow by directly desthiobiotinylating a photoaffinity probe, omitting these additional alkyne functionalization steps. Here, we compare the suitability of desthiobiotin and alkyne modified Ribocil-derived photoaffinity probes for chemotranscriptomic profiling. Our results demonstrate binding of both photoaffinity probes to their specific target, the FMN riboswitch, using *in vitro* transcription/translation and RT-qPCR. We also observed high unspecific interactions due to proposed weak and non-specific binding of the desthiobiotin moiety to RNA analyzed by dot blots and RT-qPCR. Finally, transcriptome-wide sequencing confirmed the unselective interaction of desthiobiotin. These findings suggest that desthiobiotin is an inefficient enrichment handle for the design of photoaffinity probes, resulting in many off-target interactions.

## Abstract 2

The suitability of desthiobiotin and alkyne modified Ribocil photoaffinity probes for chemotranscriptomic profiling were compared. Desthiobiotin appears to unselectively interact with RNA, rendering it unfavorable for transcriptome-wide studies.

## Graphical abstract

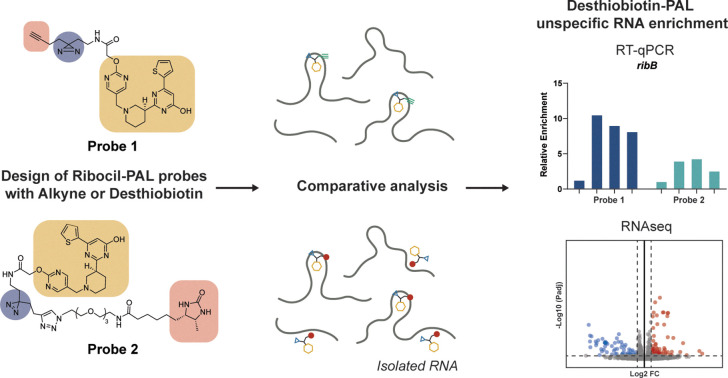

## Introduction

Advancing the understanding of interactions between small molecules and RNA is imperative for developing RNA-targeting therapeutics. Currently, most therapeutic small molecules target proteins, while they only make up 1.5% of the human genome, and the druggable proteome is even smaller.^1,2^ By shifting the focus towards RNA as therapeutic targets, we can potentially increase the number of druggable sites significantly. In particular, non-coding RNAs (ncRNAs) have been suggested as attractive targets including microRNAs (miRNAs), small nucleolar RNAs (snoRNAs) and long non-coding RNAs (lncRNAs), since they can regulate expression of disease-related genes and can form targetable conformations.^3,4^

These conformations can entail complex tertiary structures, including hairpins, pseudoknots, internal loops or bulges that can be recognized by small molecules.^5–9^ As bacterial and viral RNA are essential for the life cycles of different human pathogens, they are, therefore, interesting drug targets. In 1944, streptomycin was discovered as an antibiotic, which was later identified as the first small molecule to target bacterial ribosomal RNA (rRNA).^10^ Since that discovery many more antibacterial and antiviral RNA-targeting small molecules have been identified. Recently, many efforts were made to investigate the pseudoknot-1 PRF, essential in beta-coronaviruses like SARS-CoV-1 and SARS-CoV-2, and to identify compounds targeting this pseudoknot.^11^ An interesting class of bacterial RNAs was discovered in the early 2000s, that is able to undergo structural changes in conformation in the presence of small molecules.^12^ These riboswitches are located in the 5' untranslated region (UTR) of mRNA and thus regulate a key aspect of the bacterial life cycle. It was later discovered that the natural product antibiotic Roseoflavin inhibits riboflavin biosynthesis by binding to the flavin mononucleotide (FMN) riboswitch, highlighting riboswitches as interesting drug target.^13,14^

To assist in the investigation of small molecule–RNA interactions, there has been an increased interest in the development of chemical probes that directly report on target binding. Chemical probes can be designed by combining a small molecule of interest with a cross-linking moiety to allow for photoaffinity labeling of target RNAs and a purification tag, like biotin, to isolate RNA by pulldown.^15–17^ Early examples of this affinity-based probing include seminal work by the group of Disney who designed a small molecule that interacts with and covalently binds to the CUG trinucleotide repeat associated with myotonic dystrophy type 1 (DM1).^18,19^ When combined with next-generation sequencing (NGS), this approach allows for chemotranscriptomic screening to identify novel targets for small molecules of interest, like the precursor of the oncogenic microRNA 21 or the oncogene QSOX1.^20–22^ Besides the usefulness of this technique as a screening method, it can also be applied as a validation method to investigate the interaction of small molecules with known RNA targets.^23–27^ Interestingly, an already existing drug inhibiting the receptor tyrosine kinase was identified as an RNA targeting molecule with the help of computational modelling and validated this interaction by photoaffinity labeling.^23^ It has also been shown that this method is an effective tool to investigate riboswitches.^28–30^ Balaratnam *et al.* showed that a PreQ1 riboswitch specific chemical probe can be used to probe the human transcriptome to identify PreQ1 aptamer-like sequences.^28^

To support these ongoing endeavors we were interested to optimize the chemotranscriptomic profiling workflow. So far, most developed probes bear an alkyne handle that after photocrosslinking is modified with biotin through a Copper-catalyzed Azide–Alkyne Cycloaddition (CuAAC) and subsequent purification. We found this two-step process to be inefficient, resulting in 56% loss of yield (SI Fig. S1), as compared to direct biotinylated probes. We therefore speculated that biotinylated affinity probes could be used to potentially circumvent this enrichment inefficiency.^31^

**Fig. 1:**
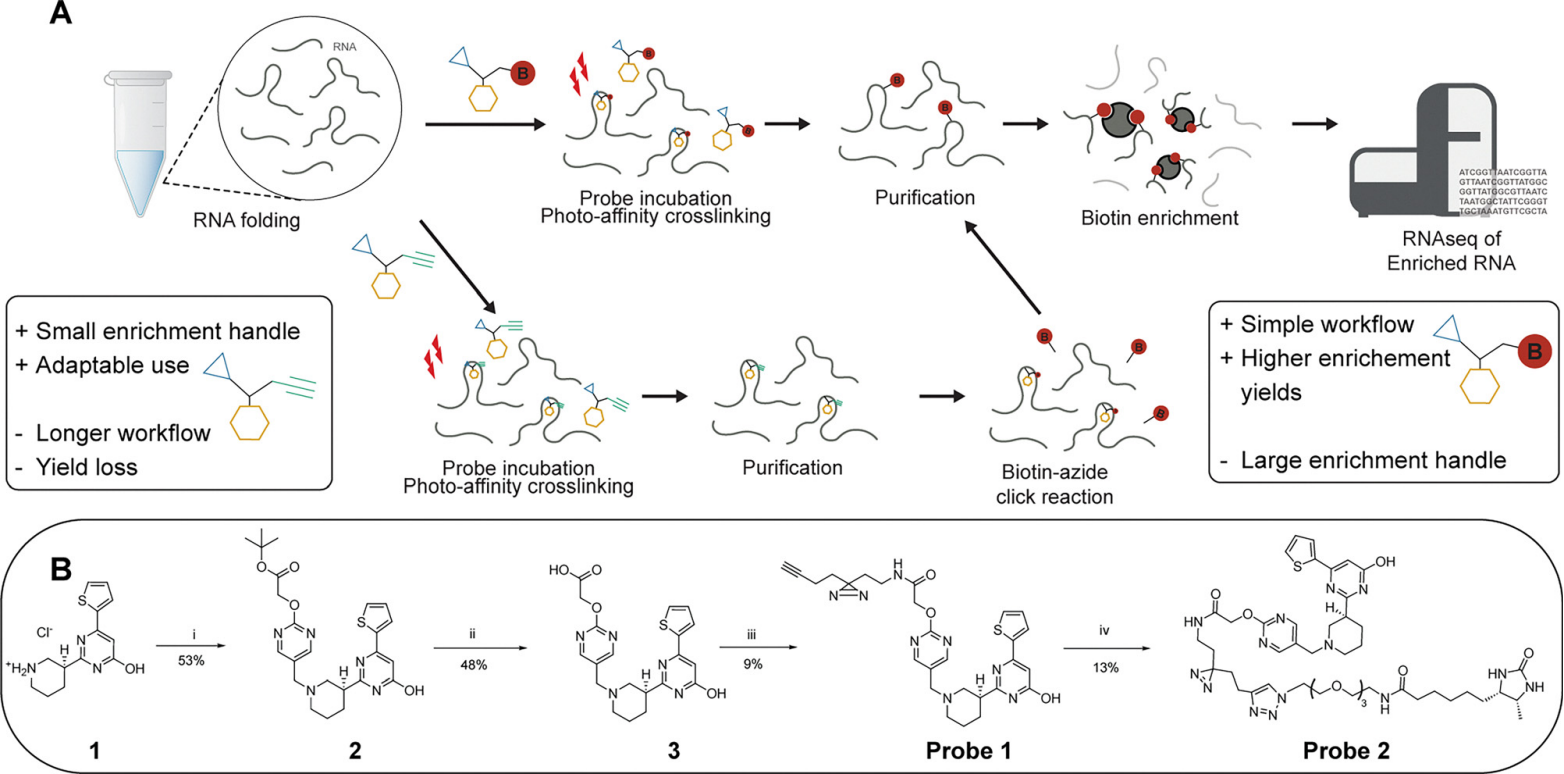
Chemotranscroptomic profiling workflow. (a) Schematic depiction of the *in vitro* chemotranscriptomic profiling workflow using chemical probes with a biotin or alkyne tag. (b) Synthesis of Ribocil-derived photoaffinity probes 1 and 2.

**Fig. 2:**
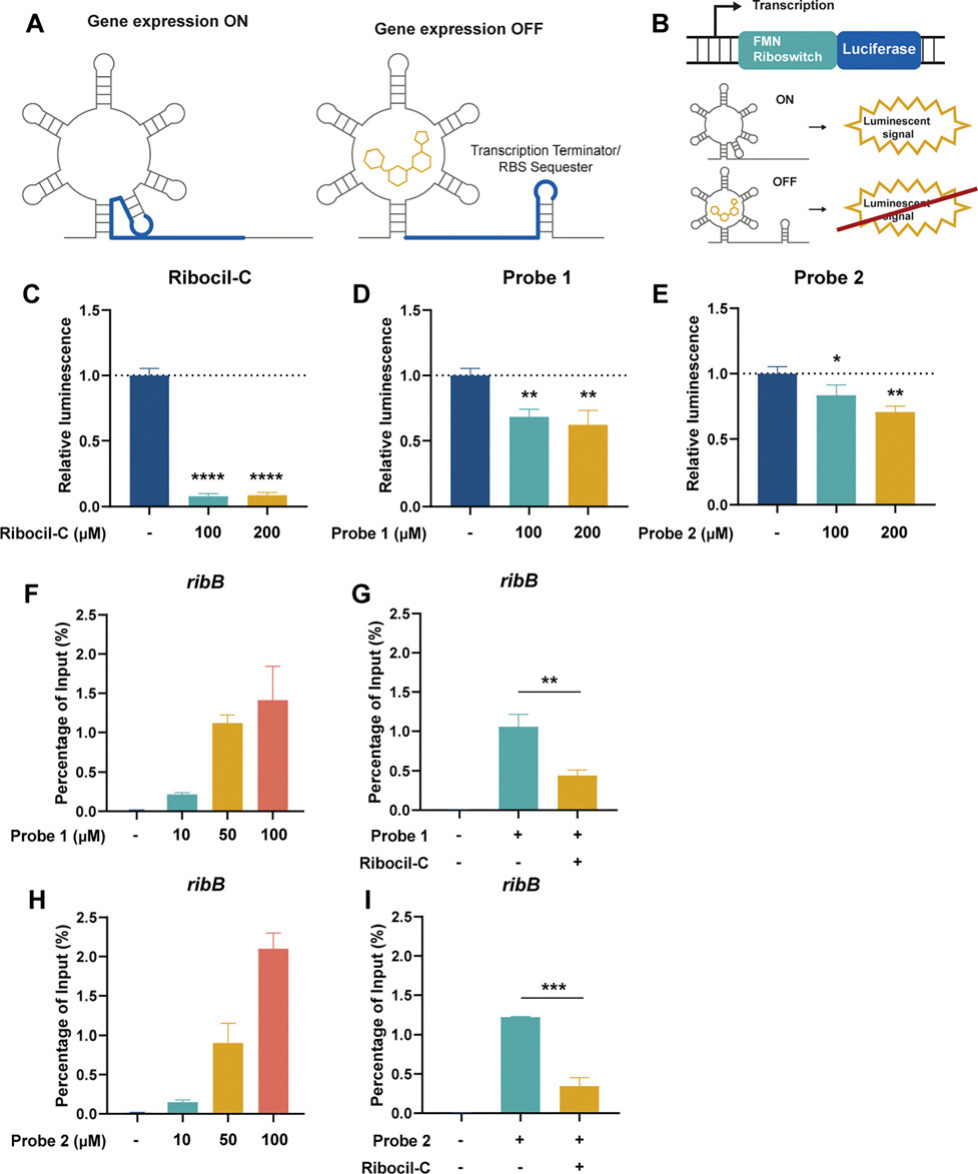
Validation of the target specificity of the Ribocil-derived probes. (A) Schematic visualization of the FMN riboswitch showing the ON and OFF conformations. (B) Schematic illustration of the IVTT assay. (C) Relative luminescence of Ribocil-C, (D) Ribocil-derived Probe 1, (E) Ribocil-derived Probe 2. (F) Enrichment of *ribB* in percentage of input at 10, 50 or 100 µM of probe 1 compared to DMSO control. (G) Competition assay showing *ribB* enrichment at 100 µM of probe 1 and Ribocil-C. (H) Enrichment of *ribB* of probe 2. (I) Competition assay of probe 2 with Ribocil-C at 100 µM. Error bars indicate standard deviations of triplicate measurements. Statistical significance was defined using an unpaired two-tailed Student's *t*-test (*p < 0.05, **p < 0.01, ***p < 0.001).

Here, we compare the suitability of desthiobiotin, a compact biotin derivative, and alkyne modified photoaffinity probes for chemotranscriptomic profiling (Fig. 1a). For this purpose, we focused on the established small molecule–RNA interaction between Ribocil and the flavin mononucleotide FMN riboswitch (Fig. 2A).^32^ Recently, we have demonstrated that Ribocil derived small molecules can be used to specifically target and investigate the FMN riboswitch.^33^ In the current study, we designed two photoaffinity probes based on the molecular structure of Ribocil bearing an alkyne or desthiobiotin moiety and validated their affinity for the FMN riboswitch. Using an *in vitro* transcription/translation (IVTT) assay and RT-qPCR we found that both Ribocil-derived photoaffinity probes are able to bind to and enrich their target. When comparing selectivities by RT-qPCR, we found the desthiobiotin probe to enrich significantly more background RNA. Combining pulldown enrichments with NGS allowed us to perform chemotranscriptomic profiling, which confirmed unselective interactions of the desthiobiotin Ribocil probe within the transcriptome. These unselective interactions were similar for a control desthiobiotin probe without the Ribocil affinity group. Taken together, we conclude that the alkyne photoaffinity probe is superior for chemotranscriptomic experiments compared to desthiobiotinylated probes.

## Results and discussion

### Synthesis and evaluation of Ribocil derived photoaffinity probes

The design of alkyne probe 1 and desthiobiotin probe 2 was inspired by the Ribocil-D scaffold.^34^ The enantiopure ribocil core 1 was prepared according to reported procedures and reacted with an aldehyde bearing pyrimidine functionalized with a *tert*-butyl ester using a reductive amination (Fig. 1b).^35^ After ester cleavage, the resulting carboxylic acid was amidated with an amine bearing diazirine/alkyne compound yielding probe 1. Further functionalization of probe 1 with desthiobiotin azide using CuAAC afforded probe 2.

To test if the synthesized probes were able to bind to the FMN riboswitch, we conducted an IVTT luciferase reporter assay. In this system, the *E. coli* FMN riboswitch controls the expression of a luciferase protein (Fig. 2A and B).^26,36^ The positive control (Ribocil) displayed strong luciferase expression inhibition by 90% at 100 and 200 µM (Fig. 2C and SI Fig. S2a). The Ribocil derivatives probe 1 and probe 2 showed moderate inhibition of 32–38% and 27–30% inhibition, respectively (Fig. 2D and E and SI Fig. S2b, c), demonstrating target engagement. We hypothesize that these differences are potentially explained by the addition of the photoaffinity and alkyne/desthiobiotin group to the structure that might inhibit optimal binding to the FMN riboswitch by steric hindrance or disruption of key interactions. Nevertheless, the observed inhibition of luciferase expression by probe 1 and 2 do imply that the probes likely interact and bind to the FMN riboswitch.

### Specificity of Ribocil derived photoaffinity probes for the FMN riboswitch

**Fig. 3:**
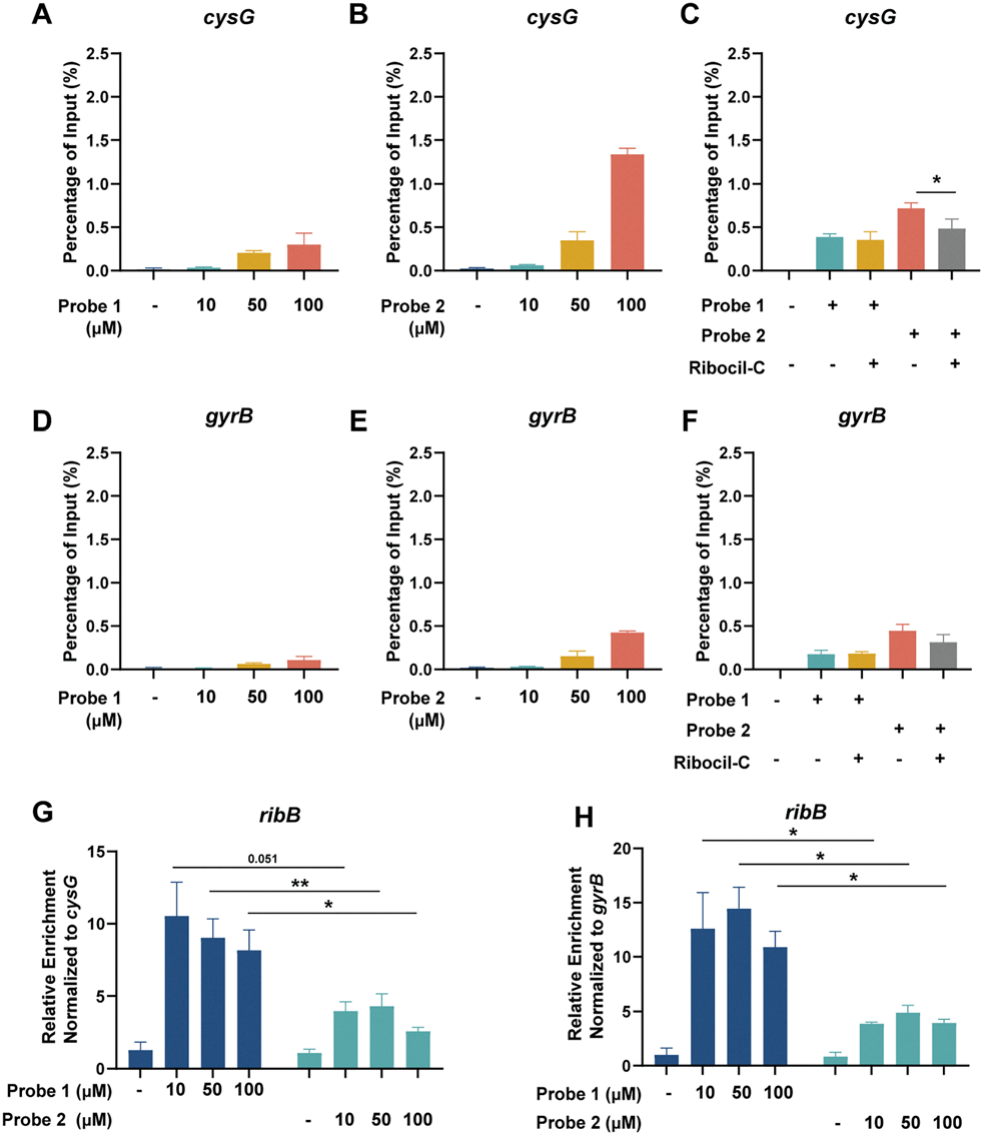
Unspecific enrichment of the Ribocil-derived probes. (A) Enrichment of *cysG* in percentage of input at 10, 50 or 100 µM of probe 1 (B) or probe 2 compared to DMSO control. (C) Competition assay showing *cysG* enrichment at 100 µM of probe 1, probe 2 and Ribocil-C. (D) Enrichment of *gyrB* in percentage of input at 10, 50 or 100 µM of probe 1 (E) or probe 2 compared to DMSO control. (F) Competition assay showing *cysG* enrichment at 100 µM of probe 1, probe 2 and Ribocil-C. (G) Relative enrichment of *ribB* normalized to *cysG* (H) or *gyrB*. Error bars indicate standard deviations of triplicate measurements. Statistical significance was defined using an unpaired two-tailed Student's *t*-test (*p < 0.05, **p < 0.01).

To investigate the specificity of the Ribocil derived probes, we incubated isolated RNA of *E. coli* with different concentrations of probes 1 and 2 and exposed them to UV light to facilitate crosslinking. To enable Streptavidin enrichment, probe 1 required an additional click-reaction with desthiobiotin-functionalized azide and subsequent purification. After Streptavidin pulldown, target RNA levels were measured using RT-qPCR and percentage (%) of input was calculated as an indication of target RNA enrichment relative to the starting material. As expected, both Ribocil derived photoaffinity probes were able to enrich the *E. coli* FMN riboswitch and its downstream gene *ribB* compared to a DMSO control in a dose-dependent manner (Fig. 2F and H). Probe 1 enriched 0.22 ± 0.02, 1.12 ± 0.10 or 1.41 ± 0.43 of *ribB* compared to probe 2 0.15 ± 0.03, 0.90 ± 0.25 or 2.10 ± 0.20% of input when treated with 10, 50 or 100 µM of each probe respectively. In contrast, DMSO treated samples showed *ribB* levels of 0.01 ± 0.005% of the input RNA compared to before pulldown. The two lower probe concentrations show slightly higher levels of *ribB* when treated with the alkyne probe 1. Importantly, at a concentration of 100 µM the desthiobiotin probe 2 appears to enrich more *ribB* than probe 1. We speculated that this increased enrichment might be explained by unspecific interactions of probe 2 with RNA. To analyze this, we also measured the levels of two housekeeping genes *cysG* and *gyrB* that are not known to interact with Ribocil.^37,38^ We observed an enrichment of 0.03 ± 0.008, 0.21 ± 0.03 or 0.30 ± 0.13% of input at 10, 50 or 100 µM of alkyne probe 1, respectively, compared to DMSO enrichment of 0.01 ± 0.02% of input (Fig. 3A). Treatment with desthiobiotin probe 2 resulted in substantially higher *cysG* levels of 0.06 ± 0.007, 0.34 ± 0.10 or 1.34 ± 0.07% of input compared to the alkyne version of the probe (Fig. 3B). In addition, to quantify the effect of background binding we calculated the relative enrichment of *ribB* by normalizing the RNA levels to *cysG*. Interestingly, probe 1 showed a relative enrichment of 10.5 ± 2.3, 9.0 ± 1.3 and 8.2 ± 1.4, while probe 2 had lower relative enrichments of 4.0 ± 0.6, 4.3 ± 0.8 and 2.6 ± 0.3 at 10, 50 or 100 µM (Fig. 3G). A similar pattern was observed for *gyrB* RNA levels (Fig. 3D–F and H). These findings show that both Ribocil derived photoaffinity probes are able to bind to and enrich its target *ribB* with similar efficiencies. However, when the enrichment levels are compensated for the overall RNA levels, represented by the housekeeping genes, we observe a 2.1–3.2-fold increase in relative enrichment of alkyne probe 1 compared to 2. This is likely explained by higher unspecific interactions of probe 2 with RNA. We speculate that the observed high enrichment of *cysG* and *gyrB* with probe 2, especially at 100 µM, stems from binding of the desthiobiotin moiety of the probe to off-target RNAs rather than true target engagement. Likely, the desthiobiotin group binds to RNA molecules in a weak and nonspecific manner, while the Ribocil core binds tightly to the FMN riboswitch target. The effect becomes most apparent at the highest concentration. We hypothesize that this non-specific binding could be mediated by desthiobiotin transiently interacting with RNA before being covalently captured by UV crosslinking.^39^ Alternatively, the effect could be due to increased capture efficiency, where the additional click-reaction for probe 1 results in loss of RNAs where the attached probe is not functionalized with desthiobiotin azide. However, in that case we would expect more enriched *ribB* of probe 2 compared to probe 1 at all concentrations.

We validated the target specificity using a competition experiment with Ribocil-C. Incubation with equimolar concentrations of Ribocil-C resulted in a decrease in *ribB* enrichment of 70 and 72% when treated with probe 1 or 2, respectively (Fig. 2G and H). Moreover, with probe 1 RNA levels of *cysG* and *gyrB* remained the same with or without addition of Ribocil-C, validating the target specificity of the Ribocil derived probe. In contrast, for probe 2 Ribocil-C decreased the amount of enriched *cysG* by 32% and *gyrB* by 30% (Fig. 3C and F).

### Desthiobiotin interacts with RNA

**Fig. 4:**
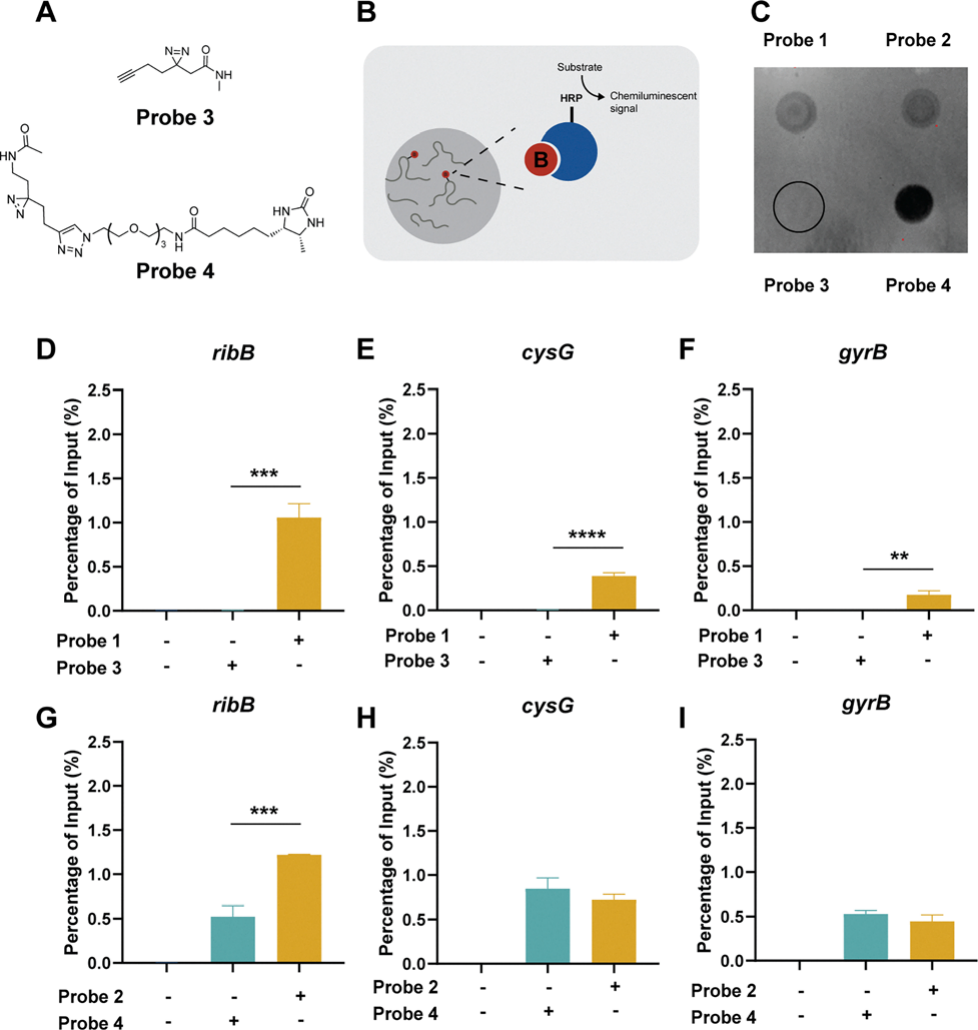
Interaction of alkyne or desthiobiotin to RNA. (a) Molecular structures of alkyne control probe 3 and desthiobiotin control probe 4. (b) Schematic depiction of the dotblot assay. (c) Dotblot membrane showing chemiluminescence. (d) Enrichment of *ribB*, (e) *cysG* and (f) *gyrB* of Ribocil-derived probe 1 and alkyne control probe 3 compared to DMSO control. (g) Enrichment of *ribB*, (h) *cysG* and (i) *gyrB* of Ribocil-derived probe 2 and desthiobiotin control probe 4 compared to DMSO control. Photoaffinity probes were incubated at a concentration of 100 µM. Error bars indicate standard deviations of triplicate measurements. Statistical significance was defined using an unpaired two-tailed Student's *t*-test (*p < 0.05, **p < 0.01, ***p < 0.001, ****p < 0.0001).

To investigate the effects of the alkyne and desthiobiotin group alone, we synthesized control probes containing only these respective functional groups and the diazirine group (Fig. 4a). The resulting control probes, probe 3,^26^ and 4, resemble the two different photoaffinity probes minus the Ribocil affinity group. Using a dot blot, we measured the total amount of desthiobiotin in RNA samples treated with the different Ribocil or control probes. We elected to focus on the 100 µM concentration, as the differences in unspecific interactions between probe 1 and 2 were most noticeable at this concentration. The two Ribocil-derived photoaffinity probes showed similar chemiluminescent levels (Fig. 4b, c and SI. Fig. S3), while the control desthiobiotin probe showed a much higher signal. In contrast, there was no signal visible for the control alkyne probe combined with an extra click-reaction to attach the desthiobiotin. Besides the dotblot analysis, we also subjected the control probes to the pulldown method. Comparable to the dotblot, probe 3 showed no signal for the measured RNA targets (Fig. 4d–f). Equal amounts of the housekeeping RNAs *cysG* and *gyrB* were enriched with desthiobiotin control probe 4*versus* desthiobiotin ribocil probe 2, but a 2.3-fold decrease was observed for the FMN riboswitch mRNA *ribB* (Fig. 4g–i). Both the dotblot and the pulldown results confirm that the desthiobiotin group by itself is able to interact with RNA, while the alkyne group does not. We cannot completely exclude the possibility that the linker between the Ribocil core and desthiobiotin is able to bind to the RNA, however. These findings suggest that desthiobiotin is likely unfavorable as an enrichment handle directly attached to photoaffinity probes for studying RNA interactions.

### Chemotranscriptomic profiling using photoaffinity probes

**Fig. 5:**
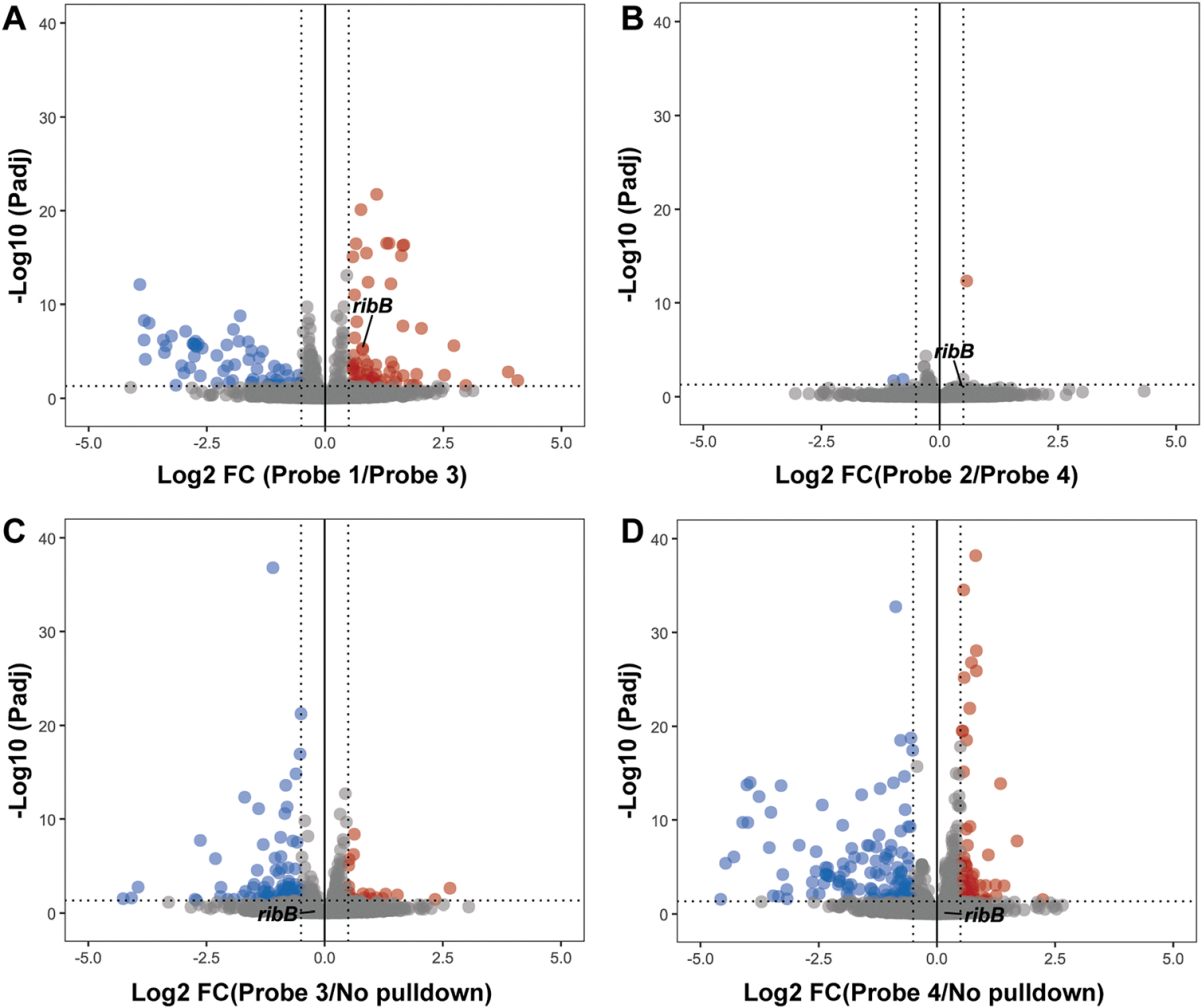
Transcriptome-wide profiling of Ribocil-derived photoaffinity probes. Volcano plots showing DESeq2 results of (A) Ribocil-derived probe 1 compared to control probe 3, (B) Ribocil-derived probe 2 compared to control probe 4, (C) control probe 3 compared to No pulldown, (D) control probe 4 compared to No pulldown. Photoaffinity probes were incubated at a concentration of 100 µM. Significant enriched genes were defined as (*P*adj < 0.05) and (0.5 < log 2 FC > 0.5). All analyses are performed on three independent replicates.

To investigate the applicability of alkyne and desthiobiotin modified photoaffinity probes for chemotranscriptomic profiling, we again performed the photoaffinity labeling and pulldown experiments and subjected enriched RNA to sequencing. We compared both Ribocil-derived photoaffinity probes to their respective control probes and analyzed enriched RNA targets. Significantly enriched RNAs were characterized by Log 2 FC > 0.5 (FC > 1.41) and *P*adj < 0.05 to distinguish small changes as well. For probe 1, we found 65 enriched targets compared to the control probe, including *ribB* at a Log 2 FC of 0.79 (Fig. 5a and SI Table S1). Interestingly, we observed only 1 enriched target for probe 2 and *ribB* was not significantly enriched compared to its control probe (Fig. 5b and SI Table S2). To understand where the discrepancy comes from, we compared probe 1 to probe 2. No large differences were observed between these two Ribocil-derived probes (SI Fig. S4). We were surprised that the sequencing data did not identify *ribB* as a hit using the desthiobiotinylated photoaffinity probe, since the RT-qPCR data showed a 2.3-fold enrichment compared to probe 4. RT-qPCR, however, is known to be more sensitive than RNA sequencing, and a 2.3-fold change might not be detected using RNA sequencing.^40^ Especially if besides target-specific interactions, the desthiobiotinylated probe also binds and crosslinks to unspecific RNAs, the actual targets are only weakly enriched. These low enrichments can be compressed by DESeq2 normalization and therefore not show up. In addition, we cannot exclude the possibility of a lack of sequencing power resulting in small differences between conditions. To keep an unbiased representation of RNA content, no ribosomal RNA depletion was performed during library preparation. To compensate, libraries were sequenced at a depth of 20 million reads for this small bacterial genome. However, for non-ribosomal RNAs this method did result in low sequencing reads (SI Table S3). We compared control probes 3 and 4 to a no pulldown condition, and as expected desthiobiotin control probe 4 showed a higher number of enriched RNA targets compared to probe 3 (Fig. 5c and d). The sequencing data confirms that the desthiobiotin might be an inefficient enrichment handle for the design of photoaffinity probes. In contrast, the alkyne photoaffinity probe did successfully identify the Ribocil target *ribB*. More research is needed to investigate the other enriched RNAs for any Ribocil-specific binding.

## Conclusions

In this study we were interested in optimizing the chemotranscriptomic profiling workflow by investigating the suitability of directly desthiobiotinylated and alkyne modified photoaffinity probes. We report here the design and validation of two Ribocil-derived photoaffinity probes. Selectivity of the two probes towards its described target, the FMN riboswitch, was determined by IVTT and RT-qPCR. Using RT-qPCR, we observed the desthiobiotin probe 2 to enrich significantly more background RNA. We speculate that the unspecific enrichment of the probe stems from weak and nonspecific binding of the desthiobiotin moiety of the probe to off-target RNAs. The unselective interactions of Ribocil probe 2 within the transcriptome were confirmed with NGS. In contrast, using transcriptome wide sequencing Ribocil probe 1 did successfully enrich its target.

Given the findings in this study, correct design of a photoaffinity probe seems crucial. In our comparative analysis using photoaffinity probes bearing the same RNA targeting moiety, we observed an increase in non-specific enriched RNAs using a directly desthiobiotinylated probe *versus* an alkyne-containing probe. Taken together, in this study we show that the desthiobiotin acts unfavourable as a direct enrichment handle in photoaffinity probes when aiming to study small molecule–RNA interactions. Although, we here only investigated one ligand–RNA interaction, we advise to be cautious when using desthiobiotin in chemotranscriptomic experiments.

## Experimental

### Synthesis of photoaffinity probes

The synthesis of Ribocil-derived and control photoaffinity probes is described in the SI (Schemes S1–S5).

### Bacterial strains and growth conditions

*E. coli* strain K12 MG1655 and TOP10 were cultured Luria-Bertani (LB) media at 37 °C in a shaking incubator. When necessary, the media was supplemented with 100 µg mL^−1^ ampicillin.

### RNA isolation

Bacterial RNA was isolated using the Quick-RNA Miniprep Kit (Zymo). Briefly, overnight bacteria cultures were diluted in LB media and grown until they reached OD_600_ 0.8. Then, the cultures were centrifuged at 8000 rpm for 5 minutes and the pellet was incubated in 100 µL Lysozyme buffer (0.4 mg mL^−1^ in 10 mM Tris–HCl, 10 mM EDTA, pH 8.0) for 20 minutes at room temperature. Cells were lysed using 600 µL RNA Lysis buffer and further purified according to the manufacturer's instructions. RNA was eluted in 50 µL nuclease-free water and concentration and purity of the RNA was determined using the NanoPhotometer N50 (Implen).

### *In vitro* transcription/translation (IVTT) assay

The FMN riboswitch controlled luciferase plasmid was isolated using the QIAprep Spin Miniprep Kit (Qiagen) using manufacturer's instructions.^26^ IVTT assays were performed using the commercially available *E. coli* T7 S30 Extract System for Circular DNA kit (Promega). The standard protocol was adapted to accommodate 11 µL reactions with 1 mM D-luciferin (Abcam Ltd) and 2 nM DNA template. IVTT reaction/DNA mixtures were mixed with 100–200 µM ligand/probe at a total volume of 11 µL, DMSO concentrations were maintained below 5%. Then 10 µL was added to a flat-bottomed white 384-wells plate (PerkinElmer) and incubated at 37 °C. Luminescence was measured every 2 minutes for 2 hours on the BioTek Synergy H1 plate reader (Agilent). Relative luminescence was calculated using the peak height of each curve.

### Photoaffinity labeling followed by streptavidin pulldown

For photoaffinity labeling, 5–10 µg of bacterial RNA was first denatured at 65 °C for 5 minutes, then snap-cooled and kept on ice. RNA was incubated with the respective photoaffinity probes or DMSO in Folding buffer (final conc. 15 mM MgCl_2_, 100 mM KCl, 50 mM HEPES, pH 8.0) at 37 °C for 30 minutes in the dark with gentle agitation. For the competition assays, RNA was first incubated with 100 µL of Ribocil-C for 30 minutes, before the photoaffinity probes were added. Samples were irradiated at 365 nm for 15 minutes using the UV stratalinker 1800 (Stratagene). After purification using ethanol precipitation, probe 1 and probe 3 were functionalized with desthiobiotin azide using a CuAAC click-reaction (final conc. 0.5 mM CuSO_4_, 5.0 mM sodium ascorbate, 2.5 mM Tris-(3-hydroxypropyltriazolylmethyl)amine and 0.1 mM desthiobiotin azide) for 1 hour at 37 °C followed by another ethanol precipitation.

For the streptavidin pulldown, 25 µL magnetic streptavidin beads (New England Biolabs) were aliquoted in a separate eppendorff tube. Purified RNA was denatured and incubated with the magnetic beads in Bead Binding buffer (150 mM NaCl, 10 mM Tris pH 7.5, 0.1% NP-40) for 1 hour rotating end-over-end at room temperature. Then, the beads underwent extensive washing, consisting of taking of the supernatant using a magnetic separator, dissolving the beads in Wash buffer and rotating end-over-end for 5 minutes. Beads were washed twice respectively using Wash buffer 1 (1% SDS), low-salt Wash buffer 2 (50 mM NaCl, 10 mM Tris, 0.1% NP-40) and high-salt Wash buffer 3 (500 mM NaCl, 10 mM Tris, 0.1% NP-40). After washing, beads were dissolved in 25 µL Elution buffer (95% formamide/H_2_O, 10 mM EDTA) and heated at 95 °C for 5 minutes. The supernatant containing the purified RNA was cleaned up using the RNA Clean & Concentrator kit (Zymo) and eluted in 15 µL nuclease-free water.

### Quantitative reverse transcription PCR (RT-qPCR)

To quantify enriched RNAs, we subjected the purified RNA to Luna Universal One-Step RT-qPCR (New England Biolabs) according to the manufacturer's protocol. Signal was measured using the QuantStudio 1 (Thermo Fisher Scientific). Primer sequences are depicted in SI Table S4. First, the 100% Input value was calculated as adjusted Ct (Input) = Ct (1% Input) − log 2(100/1). Then, the percentage of input of each target was calculated using the formula: 100 × 2^(adjusted Ct (Input) − Ct (sample)), as described before.^41^ Relative enrichment was calculated using the formula 2^−(Ct(target gene)-Ct(housekeeping gene))^.

### Dotblot assay

RNA was labeled with the Ribocil-derived or control probes as described above. The alkyne probes were further functionalized with desthiobiotin azide. Equal volume of purified RNA was loaded onto a positively charged nylon membrane (Invitrogen) and crosslinked using the UV stratalinker. Membranes were blocked using 5% BSA (Thermo Fisher Scientific) in TBST (Thermo Fisher Scientific) for 1 hour and incubated with Streptavidin-HRP in TBST (1 : 5000, Thermo Fisher Scientific) for 1 hour at room temperature with gentle shaking. Unbound streptavidin was washed away three times with TBST. Finally, the membrane was incubated in Pierce ECL Western substrate (Thermo Fisher Scientific) for 5 minutes and chemiluminescence was imaged on an iBright imaging system (Thermo Fischer Scientific). To visualize loaded RNA, the ECL substrate was washed away after imaging three times with TBST and the membrane was incubated in Methylene Blue (SERVA).

### RNA sequencing

For chemotranscriptomic profiling, RNA was sent for sequencing at the Sequencing Facility FNWI Science faculty Radboud University Nijmegen. RNA concentration was measured using the QuBit RNA High Sensitivity kit (Invitrogen). Libraries were generated by the sequencing facility using the KAPA RNA EvoPrep Kit (Roche) and sequenced using Illumina NextSeq2000 (2 × 59 bp) with a depth of 20-million reads. Sequencing reads were trimmed using Cutadapt,^42^ before aligning the reads to the *E. coli* K12 MG1655 genome using Bowtie2.^43^ The mapped reads were summarized using FeatureCounts.^44^ Enriched genes were analyzed using DESeq2. Differentially enriched genes were visualized using volcano plots and significant enriched genes were defined as (*P*adj < 0.05) and (0.5 < log 2 FC > 0.5).

## Author contributions

W. A. V. conceptualized the study. G. P. and A. D. synthesized the compounds. D. A. L. H. and G. P. performed experimental work. D. A. L. H. analyzed and visualized the data. D.A.L.H. and W. A. V. wrote the paper with input from all authors.

## Conflicts of interest

There are no conflicts to declare.

## Supplementary Material

CB-OLF-D6CB00030D-s001

## Data Availability

The supporting data has been provided as part of the supplementary information (SI). Supplementary information: RNA sequencing data for this article is available at the GEO database number related to this publication: GSE318057. See DOI: https://doi.org/10.1039/d6cb00030d.
