# Supplementary material for: Comparative analysis of alkyne- and desthiobiotinylated photoaffinity probes for chemotranscriptomic profiling
Source: RSC Chem Biol. 2026 Mar 4;7(4):641–8. doi: 10.1039/d6cb00030d (PMC12977078; doi:10.1039/d6cb00030d)
Supplement: CB-007-D6CB00030D-s001 [file CB-007-D6CB00030D-s001.pdf]

Supplementary Information (SI) for RSC Chemical Biology.
This journal is © The Royal Society of Chemistry 2026

SUPPORTING INFORMATION

Comparative Analysis of Alkyne- and Desthiobiotinylated Photoaffinity Probes for Chemotranscriptomic Profiling

Daphne A. L. van den Homberg,[a] Georgia Poulladofonou [a], Aurelia Dekens [a] and Willem A. Velema [a*]

[a]Institute for Molecules and Materials, Radboud University Heyendaalseweg 135, 6525 AJ Nijmegen

*To whom correspondence should be addressed:

E-mail: willem.velema@ru.nl

Table of contents

Supporting Figures (S1-S4) p3

Supporting Figure S1 ....p3

Supporting Figure S2 ....p4

Supporting Figure S3 ....p5

Supporting Figure S4 ....p5

Supporting tables (S1-S4) p6

Supporting table S1 ....p6

Supporting table S2 ....p6

Supporting table S3 .... p7

Supporting table S4 .... p8

Supporting table S5 .... p8

General Synthesis p9

Scheme 1....................p9

Scheme 2....................p12

Scheme 3....................p12

Scheme 4....................p14

Scheme 5....................p15

Spectra p17

References p29

Supporting Figures

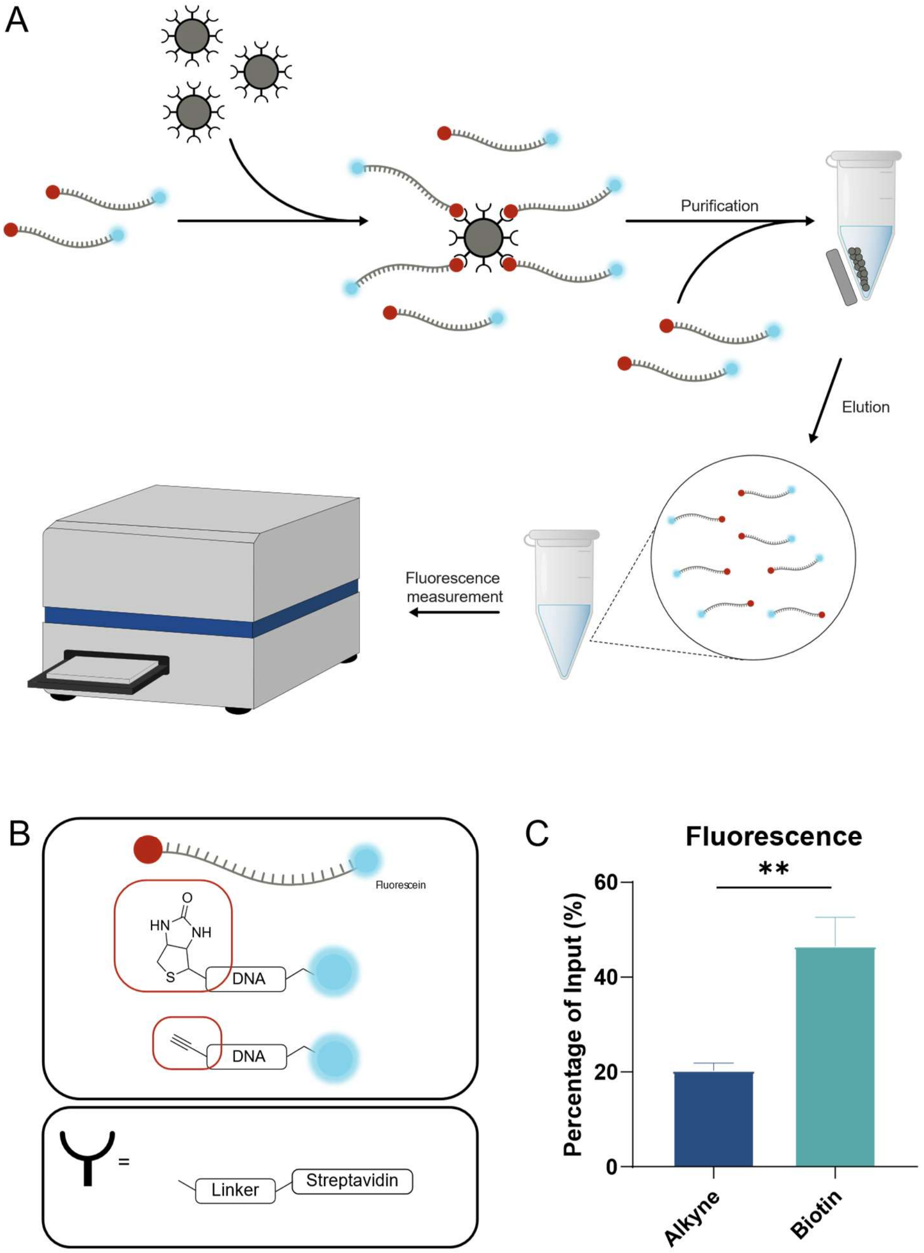

Figure S1 – Proof of concept to compare the biotin or alkyne functional group in a streptavidin pulldown. (a) Schematic visualization of the workflow using biotin- or alkyne- functionalized fluorescent DNA oligos to compare pulldown efficiencies. Alkyne containing oligos required an additional CuAAC functionalization step. Streptavidin pulldown and elution were performed as described in 'Photoaffinity labeling followed by Streptavidin pulldown'. Fluorescence of the supernatant was measured using BioTek Synergy H1 plate reader (Agilent). (b) Schematic structures of biotin and alkyne oligos, sequences can be found in supporting table 4. (c) Fluorescent signal of supernatant of enriched biotin (Biotin) or biotin-functionalized alkyne (Alkyne) DNA oligos (mean ± SD, n=3).

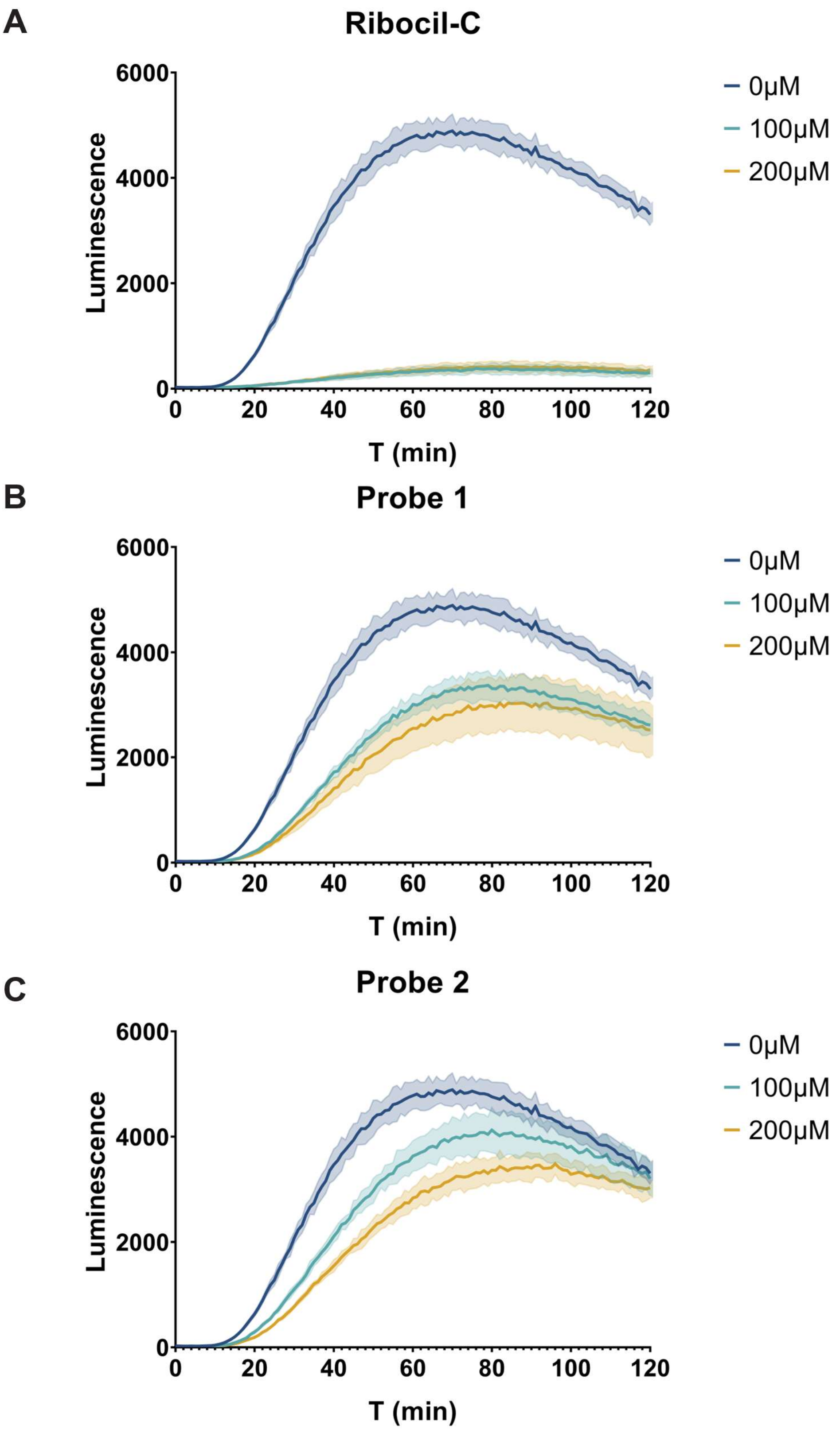

Figure S2 – Raw luminescent signal of the FMN riboswitch regulated luciferase construct inhibited by (a) Ribocil-C, (b) probe 1 or (c) probe 2 at concentrations of 100-200 μM (mean ± SD, n=3)

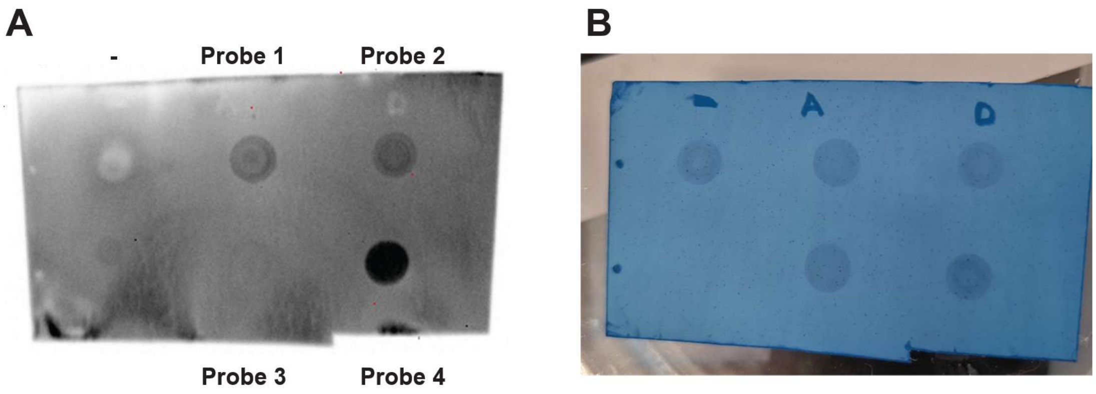

Figure S3 - (a) Dotblot membrane showing chemiluminescence, (b) Dotblot membrane showing loaded RNA stained with Methylene Blue.

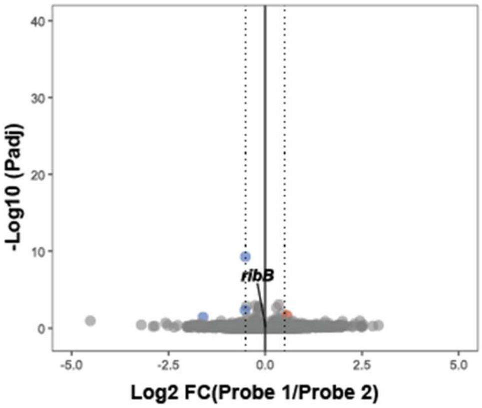

Figure S4 - Transcriptome-wide profiling of Ribocil-derived photoaffinity probes. Volcano plots showing DESeq2 results of Ribocil-derived probe **1** compared to Ribocil-derived probe **2** compared to No pulldown. Significant enriched genes were defined as (Padj < 0.05) and (0.5 < log2 FC > 0.5).

Supporting Tables

Supporting Table S1: Top 10 enriched genes of Ribocil-derived alkyne probe **1**

Gene name	baseMean	log2FoldChange	lfcSE	stat	pvalue	padj
lacY	5.669566	4.074862	1.192139	3.418109	0.000631	0.012687
arrS	11.54681	3.87522	0.96641	4.009913	6.07E-05	0.00157
pagP	4.306848	2.977649	0.997282	2.985764	0.002829	0.042877
waaS	21.73735	2.725162	0.494955	5.505875	3.67E-08	2.42E-06
rutR	12.19946	2.526127	0.664416	3.802027	0.000144	0.003372
gtrS	54.09925	2.038169	0.322935	6.311393	2.77E-10	3.44E-08
waaZ	16.57786	1.936576	0.500768	3.867214	0.00011	0.00272
ymfD	8.032104	1.932113	0.657157	2.940109	0.003281	0.048503
yoaC	12.0237	1.859763	0.613282	3.032477	0.002426	0.038809
ypeA	11.46856	1.78763	0.591438	3.022515	0.002507	0.039544

Supporting Table S2: Top 10 enriched genes of Ribocil-derived desthiobiotin probe **2**

Gene name	baseMean	log2FoldChange	lfcSE	stat	pvalue	padj
csrB	14962.58	0.571189	0.069106	8.265342	1.39E-16	4.68E-13

Supporting Table S3: Raw and FPKM sequencing counts of *ribB* and the top 10 enriched genes of of Ribocil-derived desthiobiotin probe **1** and **2**

Geneid	Probe 1 Repl1		Probe 1 Repl2		Probe 1 Repl3		Probe 2 Repl1		Probe 2 Repl2		Probe 2 Repl3		Probe 3 Repl1		Probe 3 Repl2		Probe 3 Repl3		Probe 4 Repl1		Probe 4 Repl2		Probe 4 Repl3		No Pulldown Repl 1		No Pulldown Repl 2		No Pulldown Repl 3	
	Raw	FPKM	Raw	FPKM	Raw	FPKM	Raw	FPKM	Raw	FPKM	Raw	FPKM	Raw	FPKM	Raw	FPKM	Raw	FPKM	Raw	FPKM	Raw	FPKM	Raw	FPKM	Raw	FPKM	Raw	FPKM	Raw	FPKM
ribB	370	19.29	335	24.60	336	22.99	416	22.03	409	26.78	202	16.90	238	13.62	195	12.23	258	12.65	384	14.88	300	13.77	385	18.83	265	12.54	291	14.40	384	16.43
lacY	12	0.22	12	0.31	6	0.15	4	0.08	6	0.14	4	0.12	0	0.00	2	0.04	0	0.00	6	0.08	4	0.07	4	0.07	3	0.05	12	0.21	9	0.14
arrS	22	0.20	11	0.14	7	0.08	14	0.13	9	0.10	11	0.16	0	0.00	2	0.02	1	0.01	27	0.18	6	0.05	17	0.14	22	0.18	14	0.12	20	0.15
pagP	12	0.31	6	0.22	10	0.34	6	0.16	2	0.06	0	0.00	0	0.00	2	0.06	2	0.05	6	0.12	4	0.09	4	0.10	3	0.07	3	0.07	6	0.13
waaS	33	0.75	37	1.19	21	0.63	22	0.51	22	0.63	15	0.55	2	0.05	4	0.11	10	0.21	42	0.71	29	0.58	33	0.71	14	0.29	23	0.50	26	0.49
rutR	12	0.16	10	0.19	23	0.40	8	0.11	18	0.30	11	0.23	2	0.03	4	0.06	3	0.04	17	0.17	11	0.13	14	0.17	15	0.18	18	0.22	17	0.18
gtrS	75	1.09	41	0.84	59	1.13	51	0.76	68	1.25	20	0.47	13	0.21	16	0.28	19	0.26	125	1.36	132	1.70	129	1.77	35	0.46	41	0.57	38	0.45
waaZ	32	0.60	21	0.55	18	0.44	25	0.48	19	0.45	9	0.27	2	0.04	8	0.18	11	0.19	24	0.33	28	0.46	19	0.33	11	0.19	7	0.12	20	0.31
ymfD	18	0.40	10	0.31	12	0.35	2	0.04	3	0.08	2	0.07	4	0.10	1	0.03	7	0.15	22	0.36	14	0.27	17	0.35	5	0.10	7	0.15	4	0.07
yoaC	20	0.89	13	0.82	13	0.76	14	0.63	8	0.45	4	0.29	5	0.24	7	0.38	2	0.08	10	0.33	24	0.94	16	0.67	26	1.05	11	0.47	15	0.55
ypeA	17	0.39	15	0.48	19	0.57	7	0.16	6	0.17	0	0.00	7	0.18	4	0.11	6	0.13	12	0.20	12	0.24	22	0.47	24	0.50	17	0.37	11	0.21
csrB	13872	598.11	7696	467.31	10070	569.64	17745	777.11	14716	796.65	10949	757.44	13864	656.27	12825	665.25	13918	564.42	17012	545.08	13962	530.06	12208	493.86	24363	953.68	23284	952.98	27177	961.40

Supporting Table S4: Sequences of DNA primers used for RT-qPCR

#	Short name	Sequence (5’ to 3’)
**1**	*ribB fw*	GTA ACG ATT CTG TCG GGC AT
**2**	*ribB rv*	TAC CAG AAT CAG GGC AGT CT
**3**	*cysG fw*	TTG TCG GCG GTG GTG ATG TC
**4**	*cysG rv*	ATG CGG TGA ACT GTG GAA TAA ACG
**5**	*gyrB fw*	ACCTGTTCGAGCCGATTGTT
**6**	*gyrB rv*	GCTCGCCACGTTCGATAAAC

Supporting Table S5: DNA oligonucleotides used for the proof of principle (Suppl. Fig. 1)

#	Short name	Sequence (5’ to 3’)
**1**	Biotin-oligo	/5Biosg/GTCACCTTTGGGTCACGGTCTGCTACCTTACAGGAATTCAGACCGTCCTT/36-FAM/
**2**	Alkyne-oligo	/5Hexynyl/GTCACCTTTGGGTCACGGTCTGCTACCTTACAGGAATTCAGACCGTCCTT/36-FAM/

General Synthesis

Temperatures are given in degrees Celsius (°C). Unless stated otherwise, reactions were carried out at room temperature (18-25 °C). Progress of reactions were determined using thin-layer chromatography (TLC) silica gel-coated plates (Merck 60 F254) using the described eluent. Column chromatography was performed with VWR chemicals silica gel (0.040-0.063 mm, pore size 60 Å). The separation was visualized with UV light and/or by potassium permanganate or ninhydrin staining. Chiral separation was achieved using a chiral column purchased by Daicel™ (CHIRALFLASH (TM) IA 100 x 30 mm 20 µm Preparative Column). Mass spectra (MS ESI) were recorded on a Single-Quad Thermo ISQ mass spectrometer. NMR spectra were recorded in the indicated solvent at 25 °C on a JEOL 500 MHz spectrometer equipped with a Royal HFX probe. Chemical shifts are displayed in parts per million (ppm) with respect to TMS (δ = 0.00 ppm), $CDCl_3$ (δ = 7.26 ppm) or other indicated deuterated solvent as internal reference for ^{1}H NMR; and $CDCl_3$ (δ = 77.16 ppm) or other indicated deuterated solvent as internal reference for ^{13}C NMR. Coupling constants are reported in hertz (Hz) as *J* values. The multiplicity is described by the number of coupling constants, the number of peaks, and the pattern in the signal (s=singlet, d=doublet, t=triplet, q=quartet, dd= doublet of doublets, dt=doublet of triplets, ddd=doublet of doublet of doublets, m=multiplet, br=broad). The peak assignments in ^{1}H and ^{13}C spectra are based on 2D COSY, HSQC and HMBC spectra. Each stated ^{1}H NMR, ^{13}C NMR and mass spectra result is depicted at the end of the document.

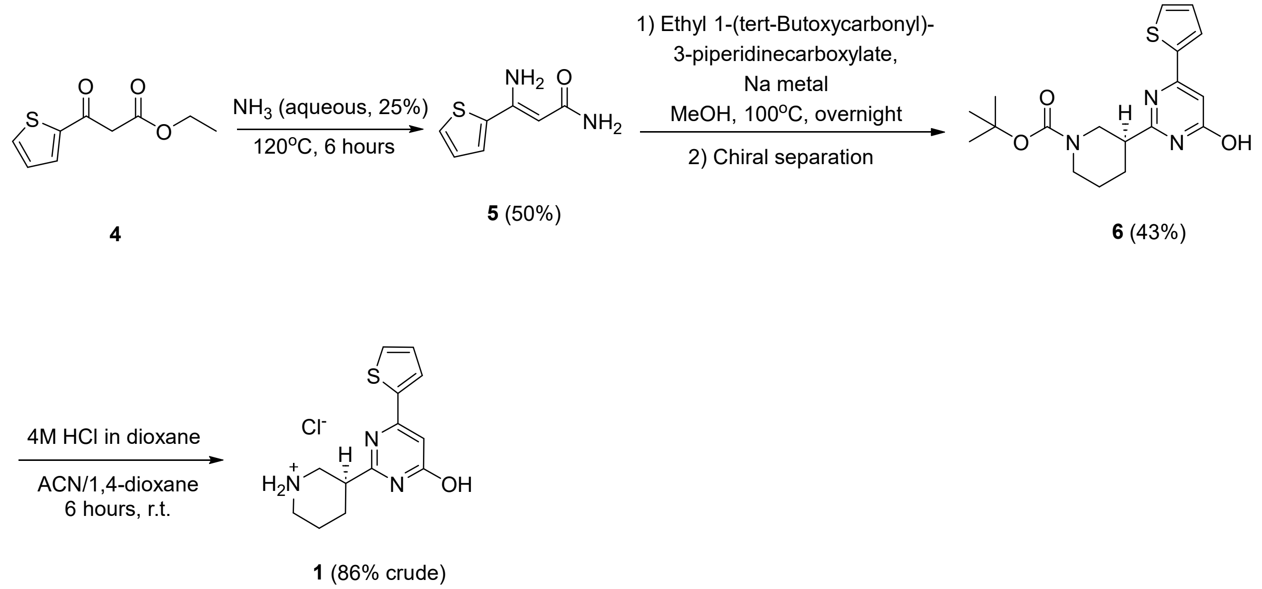

Scheme S1: Synthesis of compound **1.**

(*Z*)-3-amino-3-(thiophen-2-yl)acrylamide (**5**)

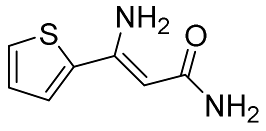

Compound **5** was synthesized according to a literature protocol.[1] In brief, ethyl 3-Oxo-3-(2-thienyl)propionate, **4** (4.6 g, 22.7 mmol, 1.0 eq.) was added to a high pressure tube and was dissolved in 25% NH_4OH (32 mL). The reaction was stirred at 120°C for 6 hours. It was then cooled to room temperature, and the solid precipitate was collected, washed with H_2O and dried under reduced pressure to give **5** as off-white solid (1948 mg, 51% crude yield). The product was used for the next step without any further purification.

^{1}H NMR (500 MHz, DMSO-*D6*) δ 7.54 (dd, J = 5.0, 1.2 Hz, 1H), 7.45 (dd, J = 3.7, 1.2 Hz, 1H), 7.08 (dd, J = 5.0, 3.7 Hz, 1H), 6.92 – 6.68 (br. s, 2H), 6.29 – 6.04 (br. s, 2H), 4.92 (s, 1H).

tert-butyl (*S*)-3-(4-hydroxy-6-(thiophen-2-yl)pyrimidin-2-yl)piperidine-1-carboxylate (**6**)

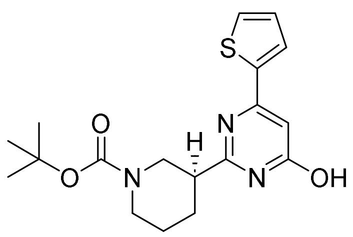

Compound **6** was synthesized according to a literature protocol.[1] In brief, Na metal (1449 mg, 63.3 mmol, 15 eq.) was added to a high pressure tube and dissolved in MeOH (20 mL). Once Na metal was fully dissolved, **5** (700 mg, 4.16 mmol, 1.0 eq.) was added to the solution along with ethyl 1-(*tert*-butoxycarbonyl)-3-piperidinecarboxylate (2162 mg, 8.40 mmol, 2.0 eq.). The reaction was heated to 100°C and stirred overnight. The solvent was then removed under reduced pressure, and the brown crystals were re-dissolved in H_2O (30 mL). The solution was neutralized to pH = 4 using HCl aqueous solution and extracted with ethyl acetate (3x). The combined organic layers were washed with brine and dried over $MgSO_4$. The crude was purified with column chromatography on silica gel (ethyl acetate : pentane, 1:1 to 4:1). The product was obtained as racemic mixture which was further separated with chiral separation (70% heptane : 30% isopropanol : 1% diethylamine) to give **6** as white solid (660 mg, 43% yield).

^{1}H NMR (500 MHz, $CDCl_3$) δ 7.67 (dd, J = 3.7, 1.2 Hz, 1H), 7.52 – 7.47 (m, 1H), 7.12 (dd, J = 5.0, 3.7 Hz, 1H), 6.62 (s, 1H), 4.36 – 4.15 (m, 1H), 4.07 – 3.98 (m, 1H), 3.30 (d, J = 18.0 Hz, 1H), 2.92 (s, 1H), 2.80 (tt, J = 10.4, 3.9 Hz, 1H), 2.15 (dd, J = 12.4, 4.5 Hz, 1H), 1.94 – 1.79 (m, 2H), 1.69 – 1.57 (m, 1H), 1.45 (s, 9H).

MS (ESI) (m/z) calculated for $[M+H]^+$:362.46, found 362.29

(*S*)-3-(4-hydroxy-6-(thiophen-2-yl)pyrimidin-2-yl)piperidin-1-ium (**1**)

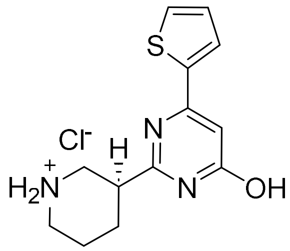

Compound **1** was synthesized according to a literature protocol.[1] In brief, **6** (296 mg, 0.819 mmol, 1.0 eq.) was dissolved in ACN/dioxane, 1:4 mixture (3.3 mL). 4M HCl in dioxane (2 mL) was added and the reaction was stirred at room temperature for 4 hours. It was then concentrated under reduced pressure to give **1** as light brown solid (254 mg, 86% crude yield). The product was used for the next steps without any further purification.

^{1}**H NMR** (500 MHz, DMSO-D_6) δ 12.49 (s, 1H), 8.97 (s, 1H), 8.88 (d, J = 10.9 Hz, 1H), 7.84 (dd, J = 3.8, 1.2 Hz, 1H), 7.74 (dd, J = 5.0, 1.1 Hz, 1H), 7.16 (dd, J = 5.0, 3.8 Hz, 1H), 6.69 (s, 1H), 3.44 (dd, J = 12.9, 3.5 Hz, 1H), 3.24 – 3.10 (m, 2H), 3.03 (tt, J = 11.2, 3.7 Hz, 1H), 2.87 (dtdd, J = 13.4, 10.3, 6.7, 2.7 Hz, 1H), 2.08 (dd, J = 12.9, 3.8 Hz, 1H), 1.84 (dt, J = 14.0, 3.7 Hz, 1H), 1.77 – 1.51 (m, 2H).

MS (ESI) (m/z) calculated for $[M+H]^+$:262.35, found 262.20

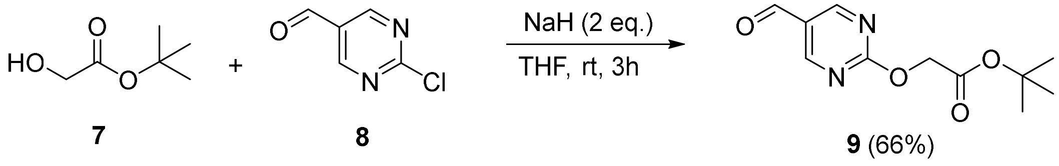

Scheme S2: Synthesis of compound **9.**

tert-butyl 2-((5-formylpyrimidin-2-yl)oxy)acetate (**9**)

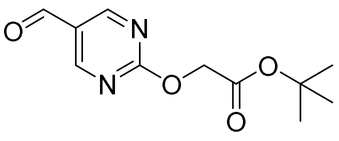

Tert-butyl-2-hydroxy acetate, **7** (2032 mg, 15.4 mmol, 2.0 eq.) was added to a two-necked round-bottomed flask under inert atmosphere. It was dissolved in dry THF (10 mL), cooled with an ice bath, and then sodium hydride (365.4 mg, 15.2 mmol, 2.0 eq.) was added, followed by 2-chloropyrimidine-5- carbaldehyde, **8** (1112 mg, 7.8 mmol, 1.0 eq.). The reaction was stirred at room temperature for 3 hours and then diluted with water. THF was removed under vacuum, and the aqueous phase was extracted with EtOAc (4x). The combined organic layers were washed with brine, dried over $MgSO_4$ and purified by column chromatography (ethyl acetate : pentane, 1 : 2) to give **9** as colorless oil (1195 mg, 66% yield).

^{1}H NMR (500 MHz, DMSO-*D_6*) δ 9.97 (s, 1H), 9.05 (s, 2H), 4.91 (s, 2H), 1.36 (s, 9H).

^{13}C NMR (126 MHz, DMSO- *D_6*) δ 190.16, 167.47, 166.54, 162.11, 125.46, 82.23, 64.97, 28.16.

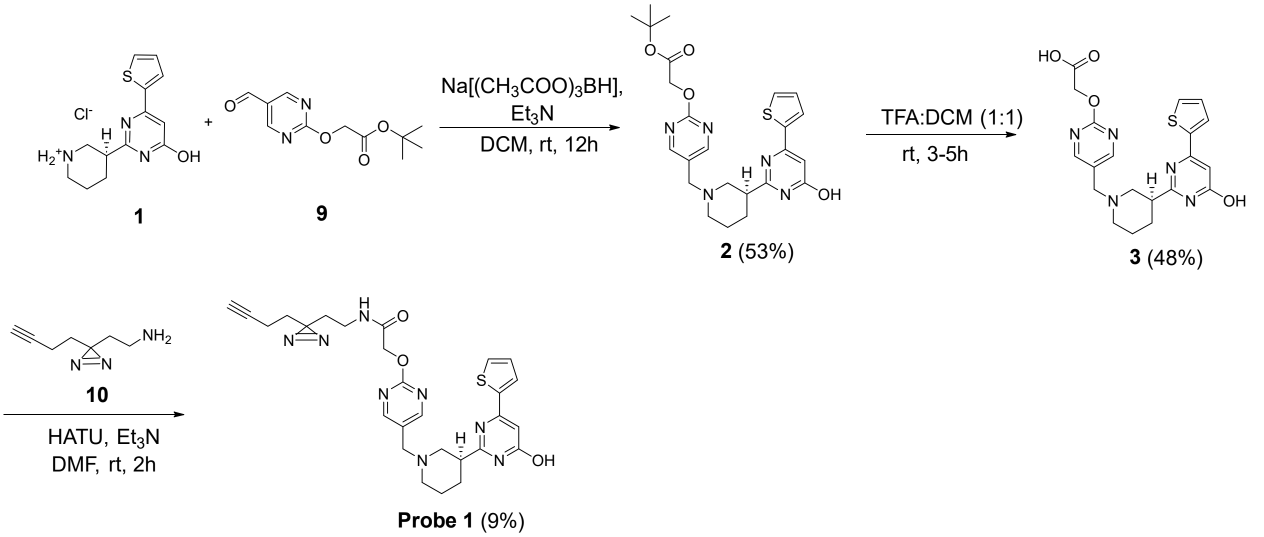

Scheme S3: Synthesis of **Probe 1** starting from compound **1.**

tert-butyl (*S*)-2-((5-((3-(4-hydroxy-6-(thiophen-2-yl)pyrimidin-2-yl)piperidin-1-yl)methyl)pyrimidin-2-yl)oxy)acetate (**2**)

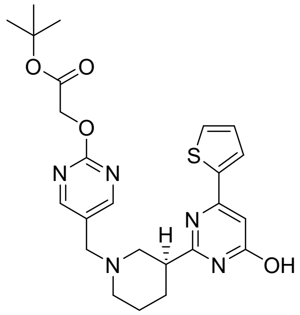

In a dried three-necked round-bottomed flask, flushed with Ar, were added activated molecular sieves and dry DCM. Then, **1** (101 mg, 0.34 mmol, 1.0 eq.), triethylamine (0.1 mL, 0.72 mmol, 2.1 eq.), **9** (81 mg, 0.34 mmol, 1.0 eq.) and sodium triacetoxyborohydride (297 mg, 1.4 mmol, 4.1 eq.) were added and the resulting light-yellow suspension was stirred at room temperature overnight. The reaction was then quenched with saturated $NaHCO_3$ aqueous solution and extracted with DCM (3x). The combined organic layers were washed with brine, dried over $MgSO_4$ and purified by column chromatography (DCM : MeOH, 95 : 5) to give **2** as yellow solid (83 mg, 53% yield).

^{1}H NMR (500 MHz, DMSO-*D_6*) δ 12.27 (s, 1H), 8.47 (s, 2H), 7.79 (dd, J = 3.7, 1.2 Hz, 1H), 7.70 (dd, J = 5.0, 1.1 Hz, 1H), 7.13 (dd, J = 5.0, 3.7 Hz, 1H), 6.60 (s, 1H), 4.76 (d, J = 1.2 Hz, 2H), 4.42 (d, J = 5.3 Hz, 1H), 3.52 (d, J = 13.6 Hz, 1H), 3.43 (d, J = 13.6 Hz, 1H), 2.92 – 2.85 (m, 1H), 2.73 (ddt, J = 10.5, 7.7, 4.1 Hz, 2H), 2.19 (t, J = 10.6 Hz, 1H), 2.04 – 1.93 (m, 1H), 1.87 (d, J = 10.2 Hz, 1H), 1.71 – 1.65 (m, 1H), 1.50 (q, J = 11.9, 11.4 Hz, 1H), 1.31 (s, 9H).

^{13}C NMR (126 MHz, DMSO- *D_6*) δ 168.04, 163.99, 160.21, 158.79, 156.83, 143.89, 130.98, 129.83, 129.21, 127.77, 125.68, 104.75, 81.00, 65.04, 59.02, 56.81, 53.18, 41.34, 29.09, 28.14, 24.09.

MS (ESI) (m/z) calculated for $[M+H]^+$: 483.59, found: 483.83.

(*S*)-2-((5-((3-(4-hydroxy-6-(thiophen-2-yl)pyrimidin-2-yl)piperidin-1-yl)methyl)pyrimidin-2-yl)oxy)acetic acid (**3**)

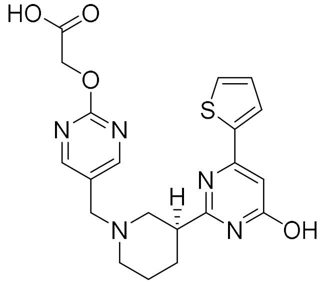

2 (251 mg, 0.52 mmol, 1.0 eq.), trifluoroacetic acid (4 mL, 52.3 mmol, 101 eq.) and DCM (4 mL) were added to a round-bottomed flask and the reaction was stirred at room temperature for 5 hours. It was then concentrated under vacuum and purified using reverse phase column chromatography (H_2O with 5% to 100% ACN, 0.1% TFA) to give **3** as white solid (137 mg, 48% yield).

^{1}H NMR (500 MHz, DMSO- *D_6*) δ 12.54 (bs, 2H), 10.16 (bs, 2H), 8.75 (s, 2H), 7.89 (s, 1H), 7.81 (d, J = 4.9 Hz, 1H), 7.21 (dd, J = 5.0, 3.7 Hz, 1H), 6.73 (s, 1H), 4.91 (s, 3H), 4.49 – 4.37 (m, 3H), 3.97 (s, 1H), 3.68 (s, 1H), 3.48 (d, J = 9.6 Hz, 1H), 3.24 (d, J = 10.5 Hz, 1H), 3.03 (d, J = 11.3 Hz, 1H), 2.13 (d, J = 10.2 Hz, 1H), 1.99 (d, J = 12.5 Hz, 1H), 1.76 (d, J = 12.4 Hz, 1H), 1.60 (d, J = 11.3 Hz, 1H).

^{13}C NMR (126 MHz, DMSO- *D_6*) δ 170.01, 169.19, 165.01, 162.77, 158.98, 158.85, 158.71, 141.84, 131.20, 129.33, 128.17, 118.42, 64.05, 63.66, 54.47, 51.85, 27.56, 22.67.

MS (ESI) (m/z) calculated for $[M+H]^+$: 428.13, found: 428.06.

(*S*)-*N*-(2-(3-(but-3-yn-1-yl)-3*H*-diazirin-3-yl)ethyl)-2-((5-((3-(4-hydroxy-6-(thiophen-2-yl)pyrimidin-2-yl)piperidin-1-yl)methyl)pyrimidin-2-yl)oxy)acetamide (**Probe 1**)

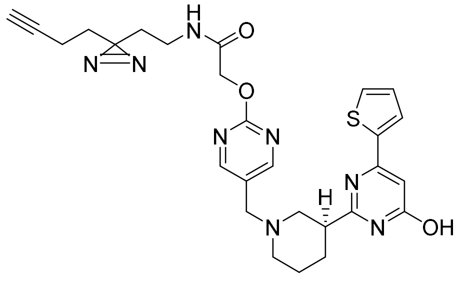

3 (59 mg, 0.11 mmol, 1.0 eq.), HATU (66 mg, 0.17 mmol, 1.5 eq.) and dry DMF (1 mL) were added to a vial stirred for 10 minutes. Then, triethylamine (0.03 mL, 0.22 mmol, 2.0 eq.) and 2-(3-(but-3-yn-1-yl)-3H-diazirin-3-yl)ethan-1-amine, **10** (15 mg, 0.11 mmol, 1.0 eq.) were added and the reaction was stirred at room temperature for 2 hours. It was then diluted with EtOAc (10 mL) and washed with saturated $NaHCO_3$ aqueous solution (3x 5 mL) and brine (3x 5 mL). The organic layer was then dried over $MgSO_4$and purified by column chromatography (DCM : MeOH, 97 : 3) to give **Probe 1** as dark brown solid (7 mg, 9% yield).

^{1}H NMR (500 MHz, $CDCl_3$) δ 12.31 (s, 1H), 8.56 (s, 2H), 7.63 (dd, *J* = 3.7, 1.1 Hz, 1H), 7.47 (dd, *J* = 5.0, 1.1 Hz, 1H), 7.11 (dd, *J* = 5.0, 3.7 Hz, 1H), 6.73 (q, *J* = 9.0, 7.4 Hz, 1H), 6.58 (s, 1H), 4.85 (s, 2H), 3.55 (d, *J* = 2.3 Hz, 2H), 3.19 (q, *J* = 6.7 Hz, 2H), 3.02 (q, *J* = 4.7 Hz, 1H), 2.89 (s, 1H), 2.72 – 2.66 (m, 2H), 2.40 (s, 1H), 2.03 (t, *J* = 2.7 Hz, 1H), 1.96 (td, *J* = 7.3, 2.7 Hz, 2H), 1.76 (qd, *J* = 9.4, 4.3 Hz, 1H), 1.67 (t, *J* = 7.0 Hz, 2H), 1.61 (t, *J* = 7.3 Hz, 2H), 1.24 (s, 2H), 0.83 (s, 1H).

^{13}C NMR (126 MHz, $CDCl_3$) δ 168.01, 163.64, 160.37, 157.47, 141.89, 129.98, 128.52, 127.26, 124.85, 105.14, 100.32, 83.01, 69.61, 66.19, 64.36, 57.20, 55.25, 53.35, 38.75, 34.14, 32.66, 32.01, 28.29, 26.76, 13.74, 13.29.

MS (ESI) (m/z) calculated for $[M+H]^+$: 547.22, found: 546.94.

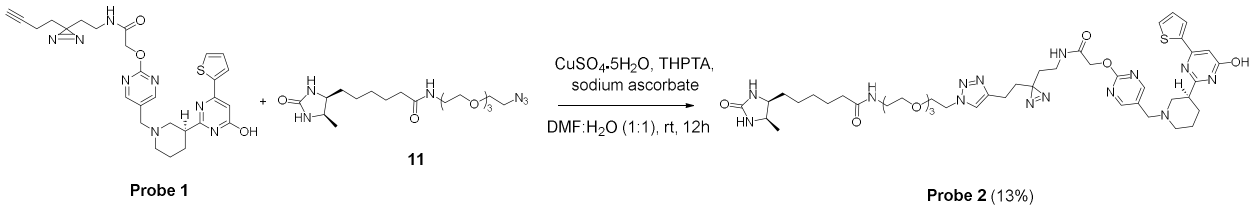

Scheme S4: Synthesis of compound **Probe 2** starting from **Probe 1.**

N-(2-(2-(2-(2-(1-(2-(3-(2-(2-((5-(((*S*)-3-(4-hydroxy-6-(thiophen-2-yl)pyrimidin-2-yl)piperidin-1-yl)methyl)pyrimidin-2-yl)oxy)acetamido)ethyl)-3*H*-diazirin-3-yl)ethyl)-1*H*-1,2,3-triazol-4-yl)ethoxy)ethoxy)ethoxy)ethyl)-6-((4*S*,5*R*)-5-methyl-2-oxoimidazolidin-4-yl)hexanamide (**Probe 2**)

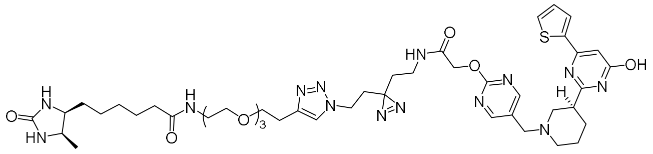

Probe 1 (8 mg, 0.015 mmol, 1.0 eq.), desthiobiotin-PEG3-Azide, **11** (15 mg, 0.036 mmol, 2.4 eq.), copper(II) sulfate pentahydrate (2.3 mg, 0.009 mmol, 0.6 eq.), sodium ascorbate (3.7 mg, 0.019 mmol, 1.2 eq.), and THPTA (1.8 mg, 0.004 mmol, 0.3 eq.) were added to a vial and suspended in DMF:H_2O, 1:1 mixture (0.13 mL). The reaction was stirred at room temperature overnight. It was then diluted with H_2O and purified by reverse phase column chromatography (H_2O with 5% to 100% ACN, 0.1% TFA) to give **Probe 2** as colorless oil (2 mg, 13% yield).

^{1}H NMR (500 MHz, DMSO- *D_6*) δ 12.49 (s, 1H), 9.76 (s, 1H), 8.69 (s, 1H), 8.09 (t, *J* = 5.8 Hz, 1H), 7.85 (s, 1H), 7.77 (d, *J* = 8.3 Hz, 3H), 7.17 (t, *J* = 4.6 Hz, 1H), 6.69 (s, 1H), 6.26 (s, 1H), 6.08 (s, 1H), 4.76 (s, 1H), 4.45 – 4.40 (m, 2H), 4.38 (d, *J* = 8.7 Hz, 1H), 3.74 (d, *J* = 5.2 Hz, 1H), 3.59 – 3.54 (m, 3H), 3.34 (t, *J* = 6.5 Hz, 2H), 3.13 (t, *J* = 5.6 Hz, 2H), 2.95 (dd, *J* = 13.8, 7.2 Hz, 3H), 2.36 (q, *J* = 8.3 Hz, 1H), 2.12 – 1.93 (m, 4H), 1.73 (d, *J* = 15.5 Hz, 1H), 1.68 (d, *J* = 7.9 Hz, 1H), 1.58 – 1.49 (m, 1H), 1.44 (dq, *J* = 21.8, 7.3 Hz, 3H), 1.29 (d, *J* = 10.5 Hz, 3H), 1.25 (s, 1H), 1.19 (dt, *J* = 13.4, 6.4 Hz, 2H), 1.13 (dd, *J* = 11.1, 5.9 Hz, 1H), 0.91 (d, *J* = 6.5 Hz, 3H).

^{13}C NMR (126 MHz, DMSO- *D_6*) δ 172.73, 167.46, 165.08, 163.33, 162.71, 159.63, 156.90, 153.13, 145.68, 131.21, 129.36, 128.19, 123.36, 70.22, 70.15, 70.08, 69.68, 69.31, 65.87, 55.50, 50.75, 49.80, 38.96, 35.76, 34.07, 32.63, 32.22, 30.04, 29.22, 26.10, 25.68, 20.67, 16.42.

MS (ESI) (m/z) calculated for $[M+H]^+$: 961.48, found: 961.29.

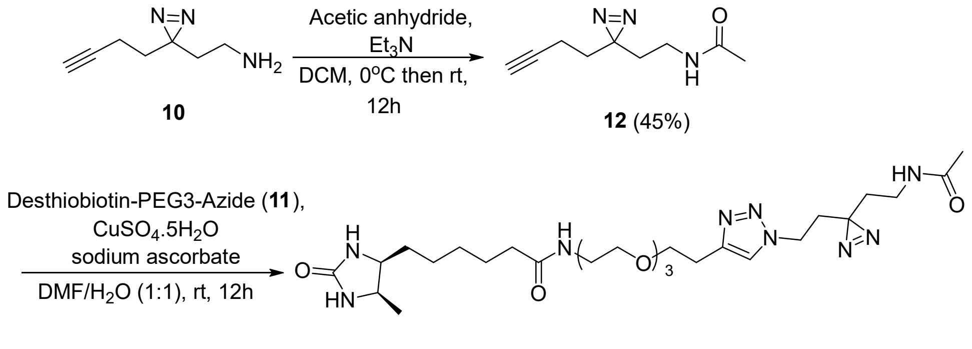

Probe 4 (35%)

Scheme S5: Synthesis of **Probe 4.**

N-(2-(3-(but-3-yn-1-yl)-3*H*-diazirin-3-yl)ethyl)acetamide (**12**)

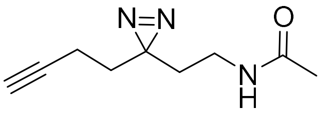

In a dried vial, flushed with Ar, was added 2-(3-(But-3-yn-1-yl)-3H-diazirin-3-yl)ethan-1-amine, **10** (39 mg, 0.262 mmol, 1.0 eq.) and dissolved in dry DCM (0.5 mL). Triethylamine (0.11 mL, 0.786 mmol, 3.0 eq.) was added and the mixture was cooled on ice before acetic anhydride (0.03 mL, 0.288 mmol, 1.1 eq.) was added dropwise. The reaction was stirred on ice, under Ar, for 10 minutes and then stirred at room temperature in the dark, overnight. It was then washed with with saturated $NaHCO_3$ aqueous solution and brine, and dried over $MgSO_4$. It was purified by column chromatography (EA/pent, 2:1) to give **12** as yellow oil (21 mg, 45% yield).

^{1}H NMR (500 MHz, $CDCl_3$) δ 5.90 (s, 2H), 3.03 (q, J = 6.5 Hz, 2H), 1.96 (d, J = 5.6 Hz, 1H), 1.92 (s, 3H), 1.68 – 1.55 (m, 6H).

^{13}C NMR (126 MHz, $CDCl_3$) δ 170.26, 82.72, 69.41, 60.41, 34.38, 32.54, 32.21, 23.23, 13.25.

N-(2-(2-(2-(2-(1-(2-(3-(2-acetamidoethyl)-3*H*-diazirin-3-yl)ethyl)-1*H*-1,2,3-triazol-4-yl)ethoxy)ethoxy)ethoxy)ethyl)-6-((4*S*,5*R*)-5-methyl-2-oxoimidazolidin-4-yl)hexanamide (**Probe 4**)

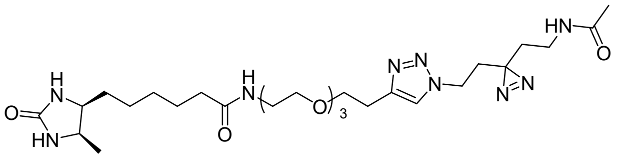

Desthiobiotin-PEG3-Azide, **11** (14 mg, 0.0332 mmol, 1.0 eq.), **12** (21 mg, 0.117 mmol, 3.5 eq.), $CuSO_4.5H_2O$ (0.8 mg, 0.00332 mmol, 0.1 eq.), and sodium ascorbate (1.4 mg, 0.00664 mmol, 0.2 eq.) were added in a small vial and suspended in DMF/H_2O, 1:1 mixture (0.17 mL). The reaction was stirred at room temperature overnight. It was then diluted with H_2O and purified with reverse phase column chromatography (H_2O with 5% to 100% ACN, 0.1 % TFA) to give **Probe 4** as a yellow oil (6.83 mg, 35% yield).

^{1}H NMR (500 MHz, $CDCl_3$) δ 7.54 (d, *J* = 12.0 Hz, 1H), 7.00 (t, *J* = 52.8 Hz, 3H), 6.52 – 6.47 (m, 1H), 4.55 – 4.49 (m, 1H), 3.99 – 3.84 (m, 1H), 3.81 (s, 2H), 3.68 – 3.51 (m, 6H), 3.44 (q, *J* = 9.3, 5.1 Hz, 1H), 3.14 – 3.03 (m, 1H), 2.50 (t, *J* = 7.3 Hz, 1H), 2.27 – 2.16 (m, 2H), 2.04 (d, *J* = 5.8 Hz, 2H), 1.88 (d, *J* = 7.2 Hz, 1H), 1.63 (dq, *J* = 12.8, 6.6, 6.1 Hz, 2H), 1.49 (qt, *J* = 12.7, 6.5 Hz, 1H), 1.43 – 1.24 (m, 5H), 1.15 (d, *J* = 6.5 Hz, 1H).

^{13}C NMR (126 MHz, $CDCl_3$) δ 173.81, 171.50, 164.49, 145.82, 122.77, 70.58, 70.48, 70.43, 70.17, 69.90, 69.46, 56.49, 51.99, 50.53, 39.36, 36.00, 34.43, 32.48, 32.10, 29.29, 28.73, 26.90, 25.90, 25.30, 22.92, 19.61, 15.59.

MS (ESI) (m/z) calculated for $[M+H]^+$: 594.73, found: 594.22.

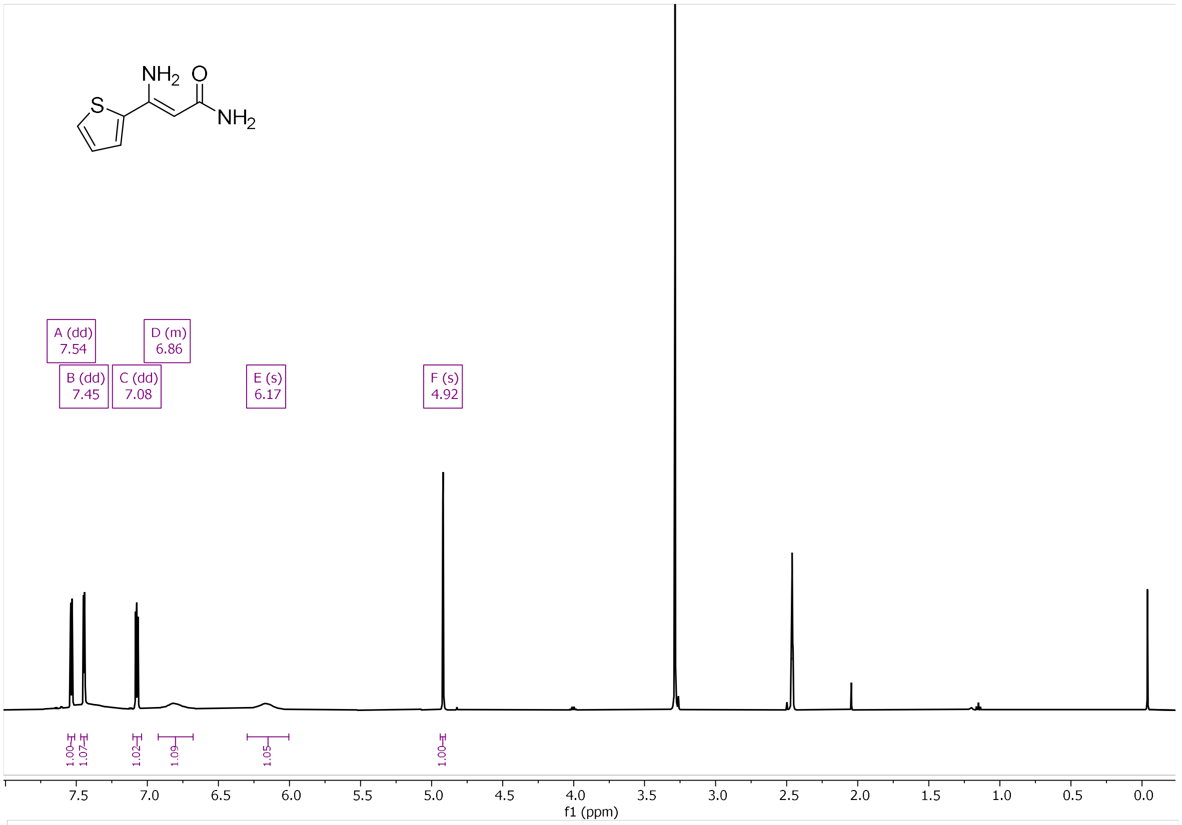
NH2
O
S
NH2
A (dd)
7.54
D (m)
6.86
B (dd)
7.45
C (dd)
7.08
E (s)
6.17
F (s)
4.92
1.00
1.07
1.02
1.09
1.05
1.00
7.5
7.0
6.5
6.0
5.5
5.0
4.5
4.0
3.5
3.0
2.5
2.0
1.5
1.0
0.5
0.0
f1 (ppm)

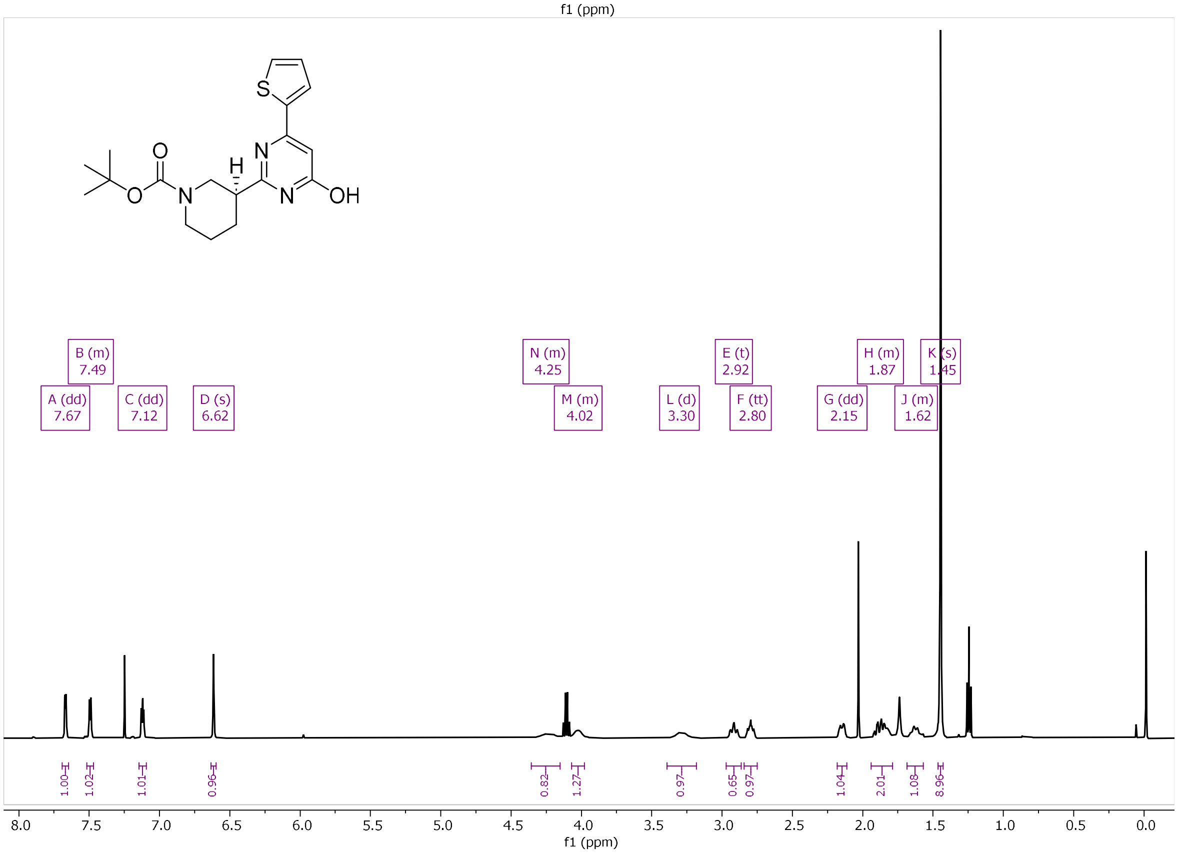
S
O
N
H
N
O
N
OH
B (m)
7.49
A (dd)
7.67
C (dd)
7.12
D (s)
6.62
N (m)
4.25
M (m)
4.02
L (d)
3.30
E (t)
2.92
F (tt)
2.80
G (dd)
2.15
H (m)
1.87
J (m)
1.62
K (s)
1.45
1.00
1.02
1.01
0.96
0.82
1.27
0.97
0.65
0.97
1.04
2.01
1.08
8.96
8.0
7.5
7.0
6.5
6.0
5.5
5.0
4.5
4.0
3.5
3.0
2.5
2.0
1.5
1.0
0.5
0.0
f1 (ppm)

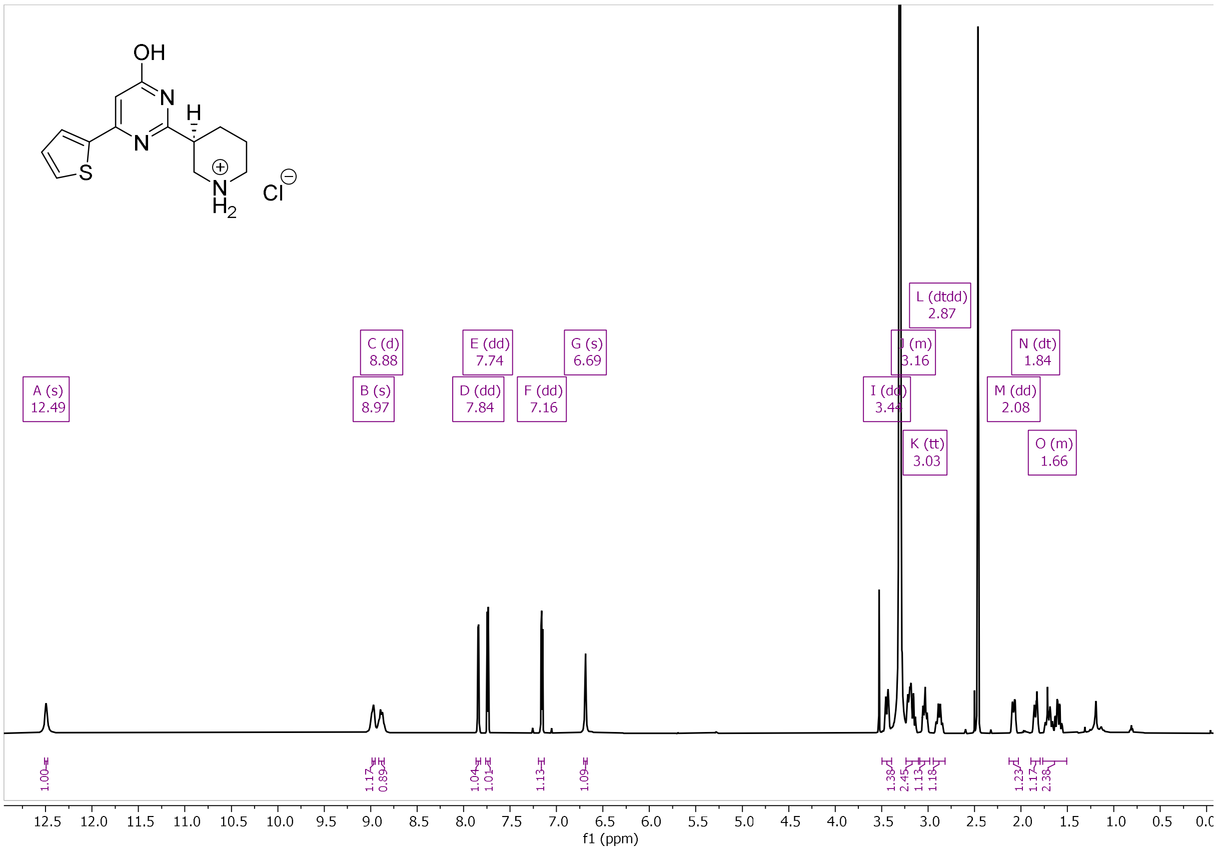

OH
N
N
H
S
N
H2
Cl
A (s) 12.49
B (s) 8.97
C (d) 8.88
D (dd) 7.84
E (dd) 7.74
F (dd) 7.16
G (s) 6.69
I (dd) 3.44
J (m) 3.16
K (tt) 3.03
L (dtdd) 2.87
M (dd) 2.08
N (dt) 1.84
O (m) 1.66
1.00
1.17
0.89
1.04
1.01
1.13
1.09
1.38
2.45
1.13
1.18
1.23
1.17
2.38
f1 (ppm)

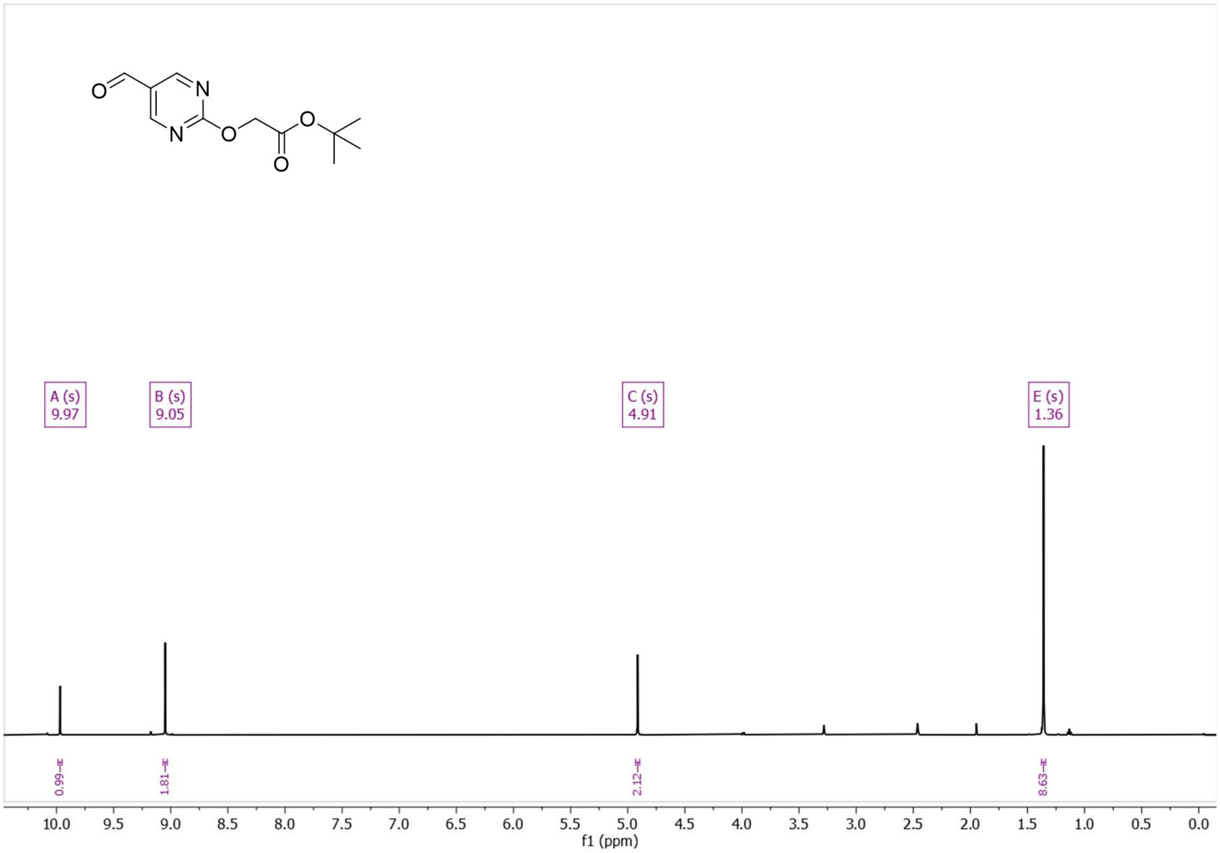
A (s)
9.97
B (s)
9.05
C (s)
4.91
E (s)
1.36
0.99
1.81
2.12
8.63
10.0 9.5 9.0 8.5 8.0 7.5 7.0 6.5 6.0 5.5 5.0 4.5 4.0 3.5 3.0 2.5 2.0 1.5 1.0 0.5 0.0
f1 (ppm)

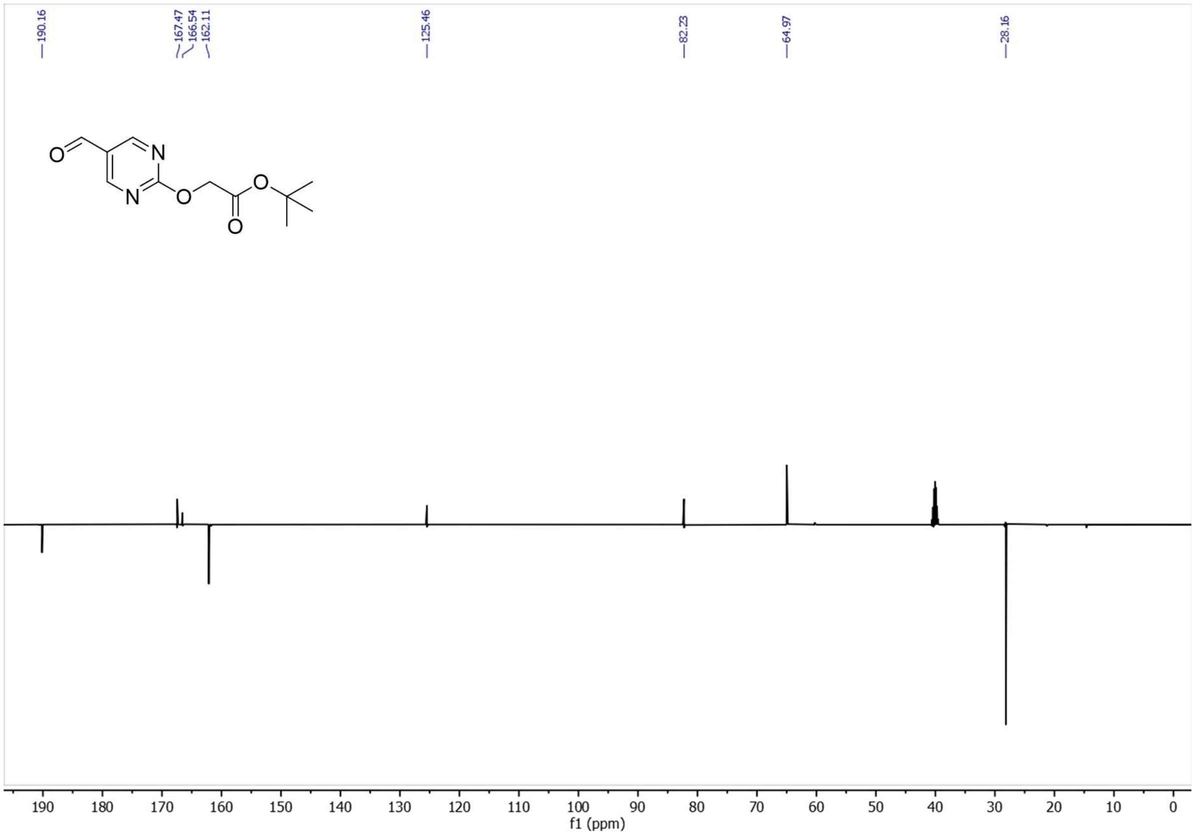
190.16
167.47
166.54
162.11
125.46
82.23
64.97
28.16
f1 (ppm)

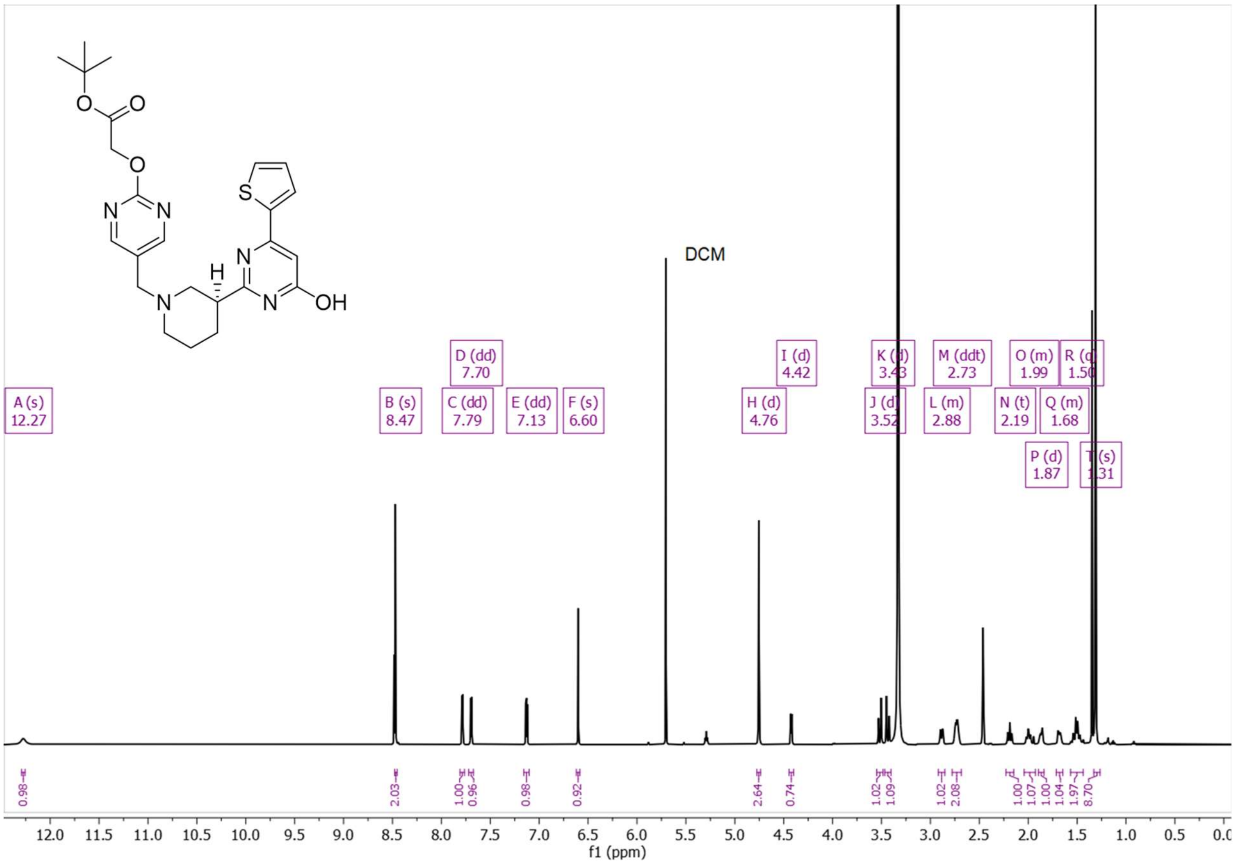
DCM
A (s) 12.27
B (s) 8.47
C (dd) 7.79
D (dd) 7.70
E (dd) 7.13
F (s) 6.60
H (d) 4.76
I (d) 4.42
J (d) 3.52
K (d) 3.43
L (m) 2.88
M (ddt) 2.73
N (t) 2.19
O (m) 1.99
P (d) 1.87
Q (m) 1.68
R (q) 1.50
T (s) 1.31
f1 (ppm)

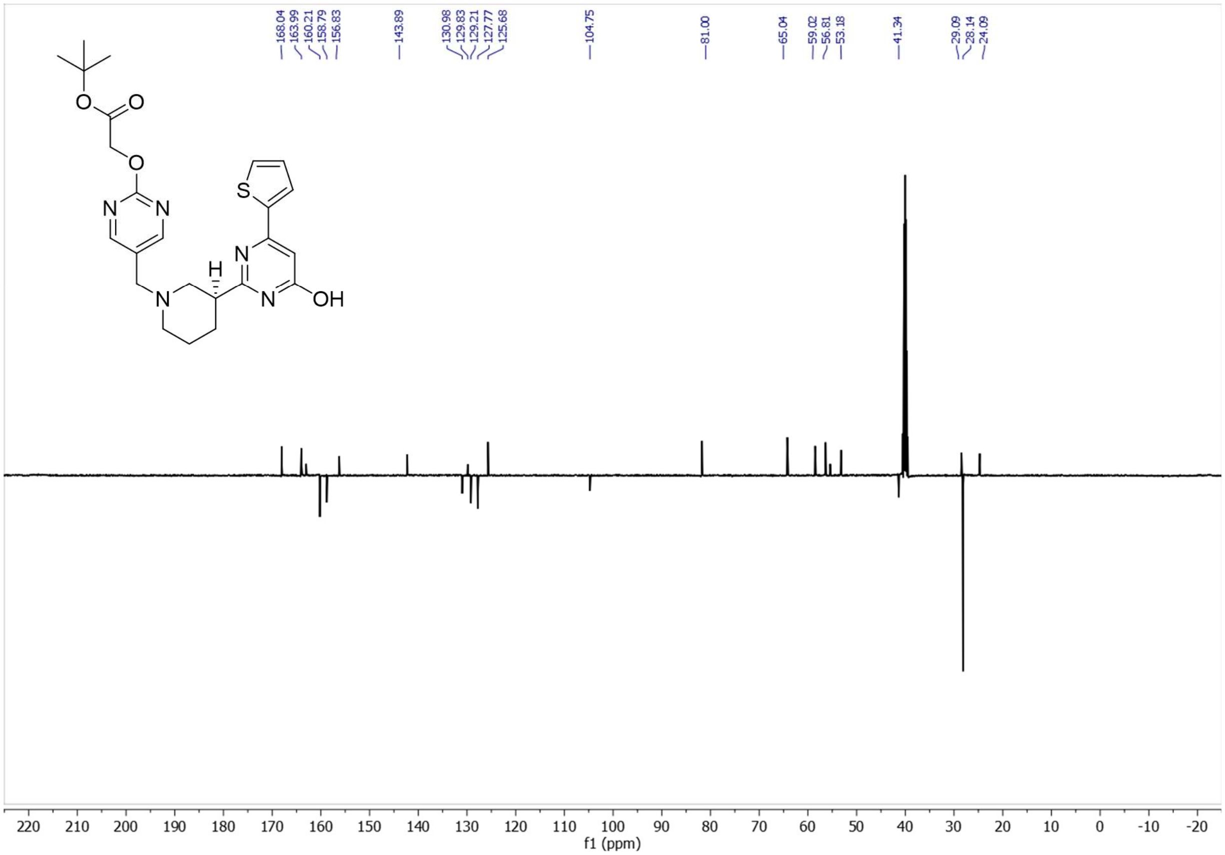
168.04
163.99
160.21
158.79
156.83
143.89
130.98
129.83
129.21
127.77
125.68
104.75
81.00
65.04
59.02
56.81
53.18
41.34
29.09
28.14
24.09
f1 (ppm)

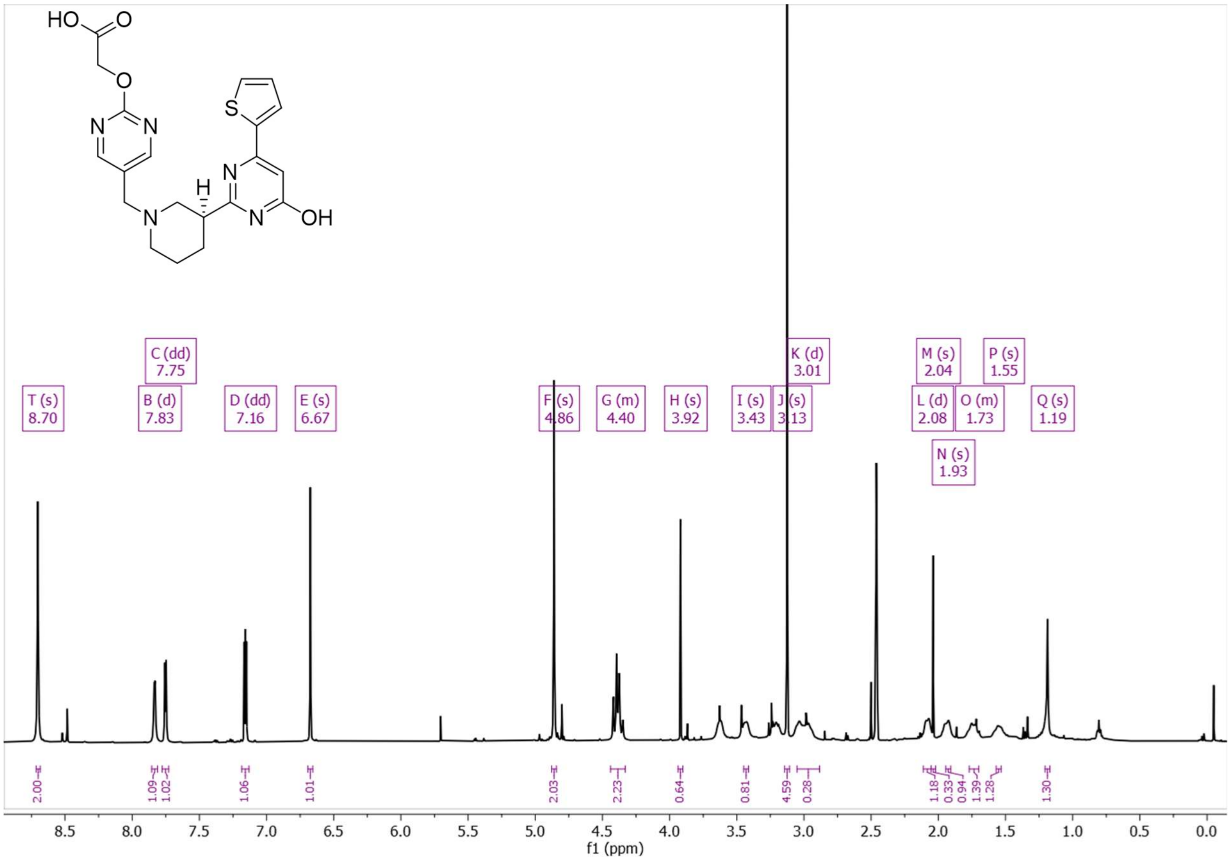
T (s) 8.70
B (d) 7.83
C (dd) 7.75
D (dd) 7.16
E (s) 6.67
F (s) 4.86
G (m) 4.40
H (s) 3.92
I (s) 3.43
J (s) 3.13
K (d) 3.01
L (d) 2.08
M (s) 2.04
N (s) 1.93
O (m) 1.73
P (s) 1.55
Q (s) 1.19
2.00
1.09
1.02
1.06
1.01
2.03
2.23
0.64
0.81
4.59
0.28
1.18
0.33
0.94
1.39
1.28
1.30
f1 (ppm)

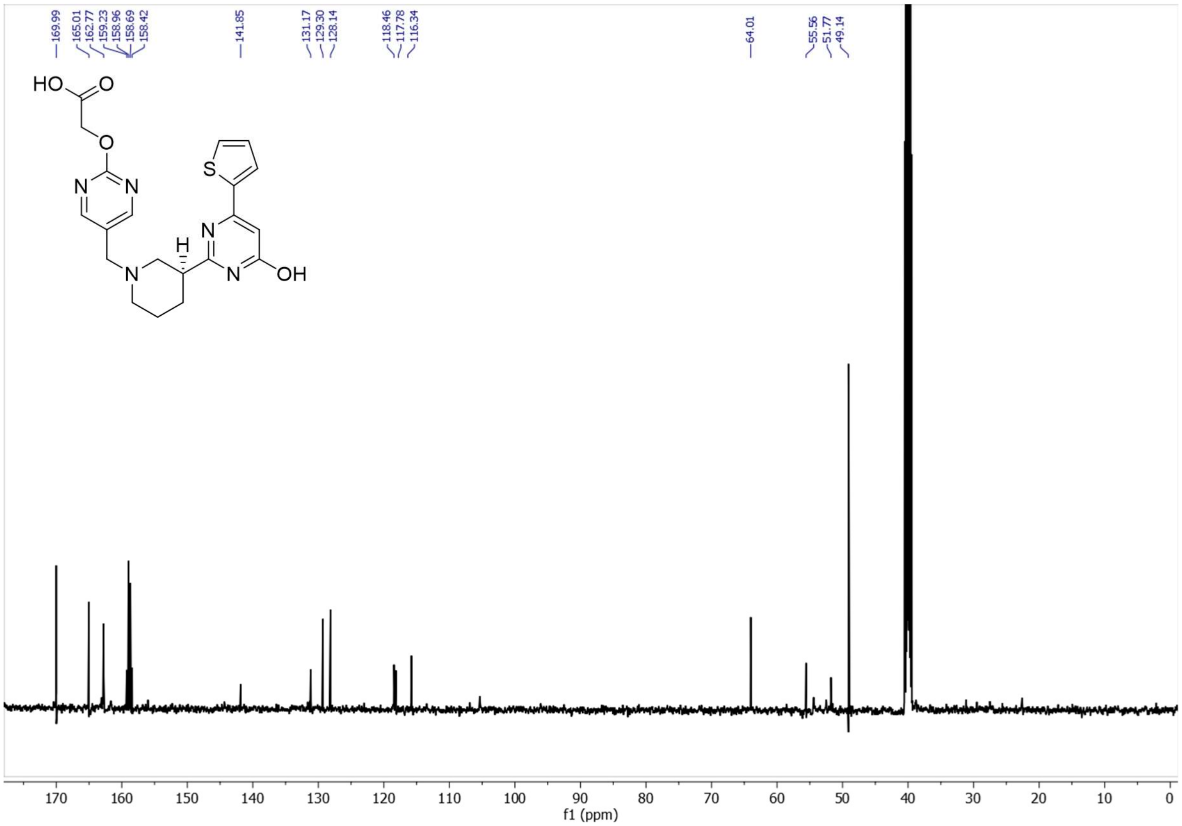
169.99
165.01
162.77
159.23
158.96
158.69
158.42
141.85
131.17
129.30
128.14
118.46
117.78
116.34
64.01
55.56
51.77
49.14
f1 (ppm)

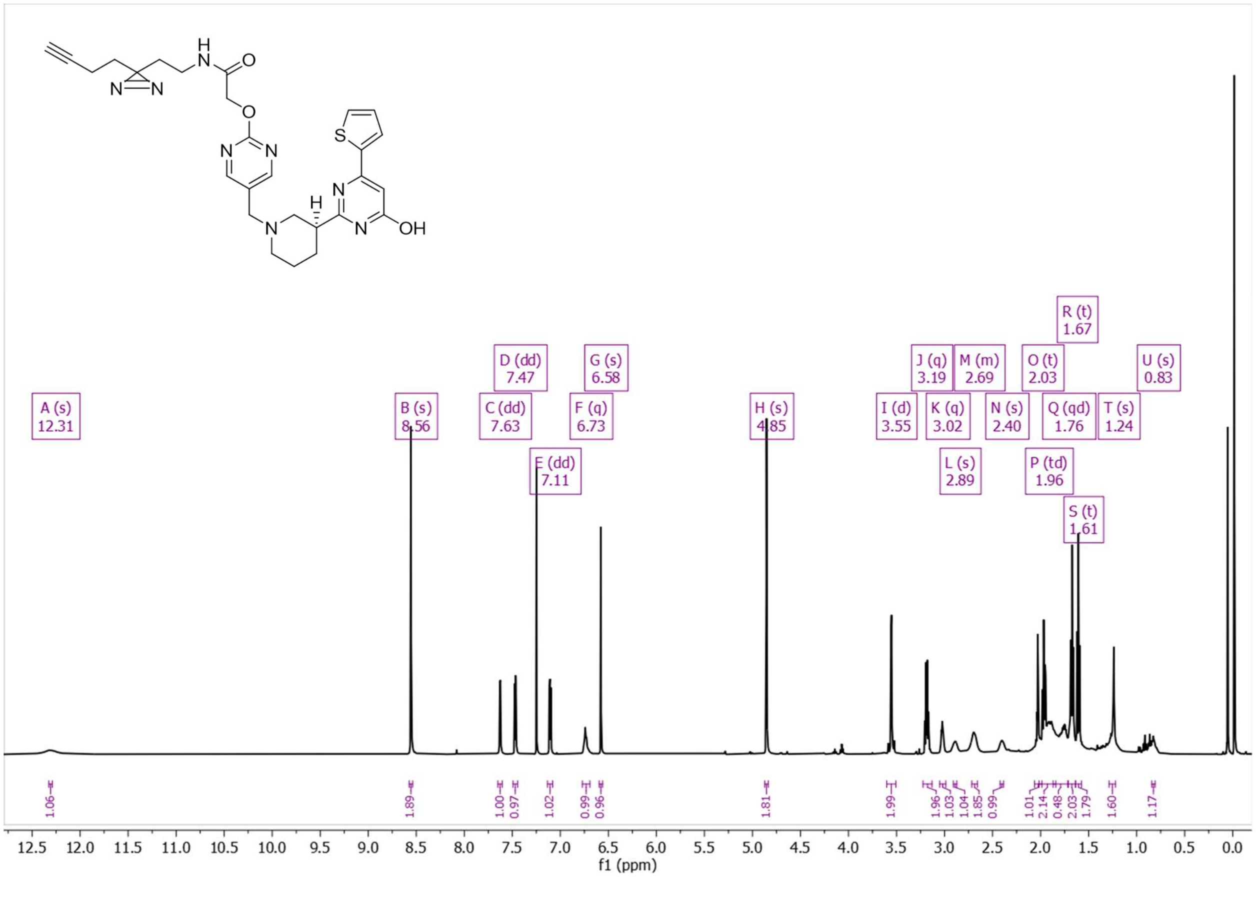
A (s) 12.31
B (s) 8.56
C (dd) 7.63
D (dd) 7.47
E (dd) 7.11
F (q) 6.73
G (s) 6.58
H (s) 4.85
I (d) 3.55
J (q) 3.19
K (q) 3.02
L (s) 2.89
M (m) 2.69
N (s) 2.40
O (t) 2.03
P (td) 1.96
Q (qd) 1.76
R (t) 1.67
S (t) 1.61
T (s) 1.24
U (s) 0.83
1.06
1.89
1.00
0.97
1.02
0.99
0.96
1.81
1.99
1.96
1.03
1.04
1.85
0.99
1.01
2.14
0.48
2.03
1.79
1.60
1.17
f1 (ppm)

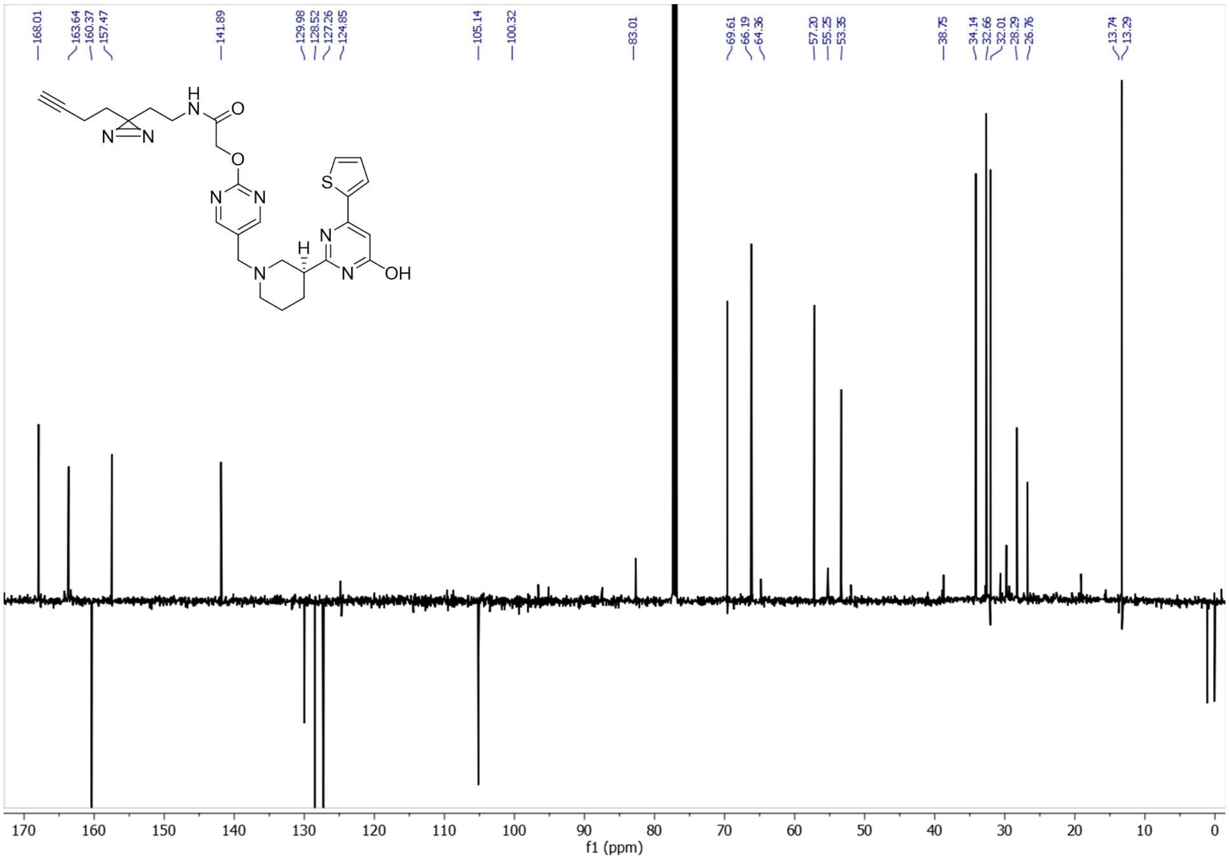
168.01
163.64
160.37
157.47
141.89
129.98
128.52
127.26
124.85
105.14
100.32
83.01
69.61
66.19
64.36
57.20
55.25
53.35
38.75
34.14
32.66
32.01
28.29
26.76
13.74
13.29
f1 (ppm)

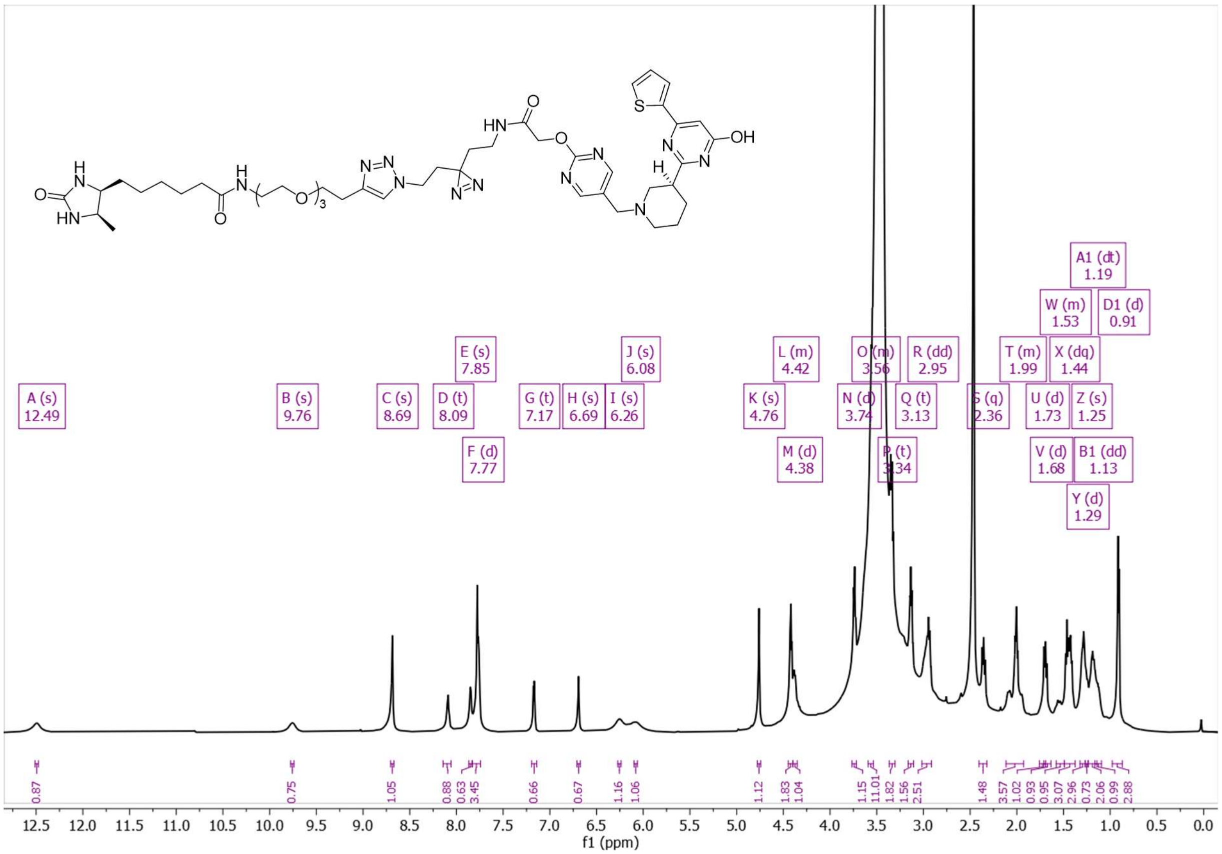
A (s) 12.49
B (s) 9.76
C (s) 8.69
D (t) 8.09
E (s) 7.85
F (d) 7.77
G (t) 7.17
H (s) 6.69
I (s) 6.26
J (s) 6.08
K (s) 4.76
L (m) 4.42
M (d) 4.38
N (d) 3.74
O (m) 3.56
P (t) 3.34
Q (t) 3.13
R (dd) 2.95
S (q) 2.36
T (m) 1.99
U (d) 1.73
V (d) 1.68
W (m) 1.53
X (dq) 1.44
Y (d) 1.29
Z (s) 1.25
A1 (dt) 1.19
B1 (dd) 1.13
D1 (d) 0.91
f1 (ppm)

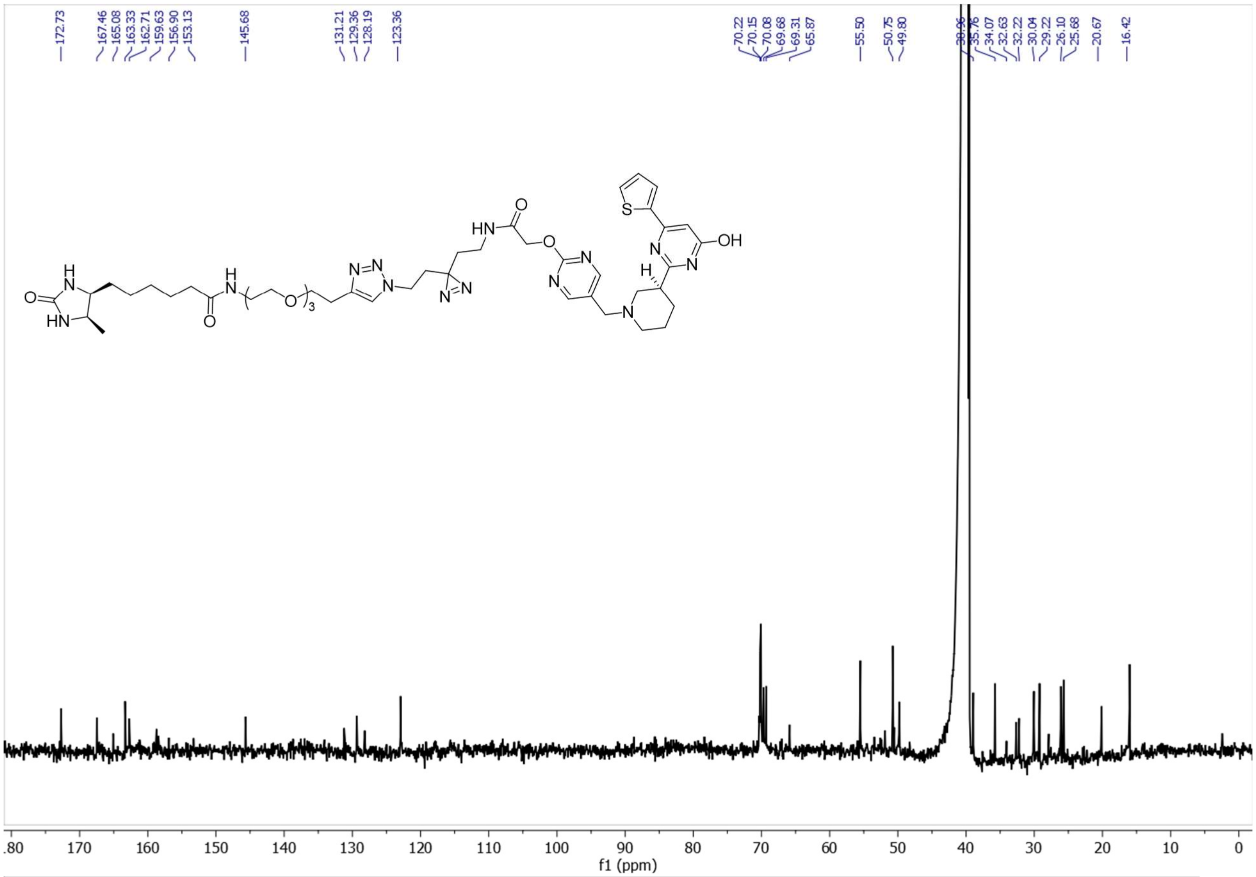
172.73
167.46
165.08
163.33
162.71
159.63
156.90
153.13
145.68
131.21
129.36
128.19
123.36
70.22
70.15
70.08
69.68
69.31
65.87
55.50
50.75
49.80
35.76
34.07
32.63
32.22
30.04
29.22
26.10
25.68
20.67
16.42
f1 (ppm)

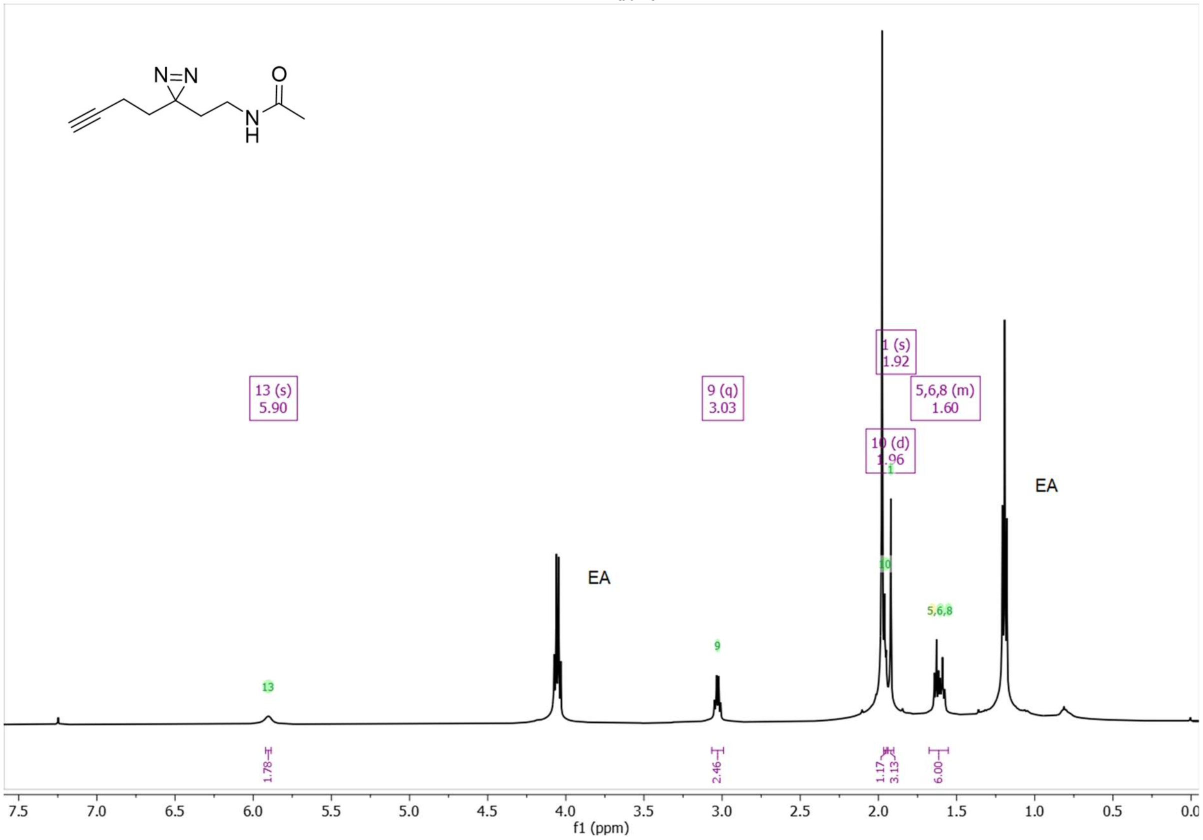
1 (s)
1.92
13 (s)
5.90
9 (q)
3.03
5,6,8 (m)
1.60
10 (d)
1.96
EA
EA
1.78
2.46
1.17
3.13
6.00
f1 (ppm)

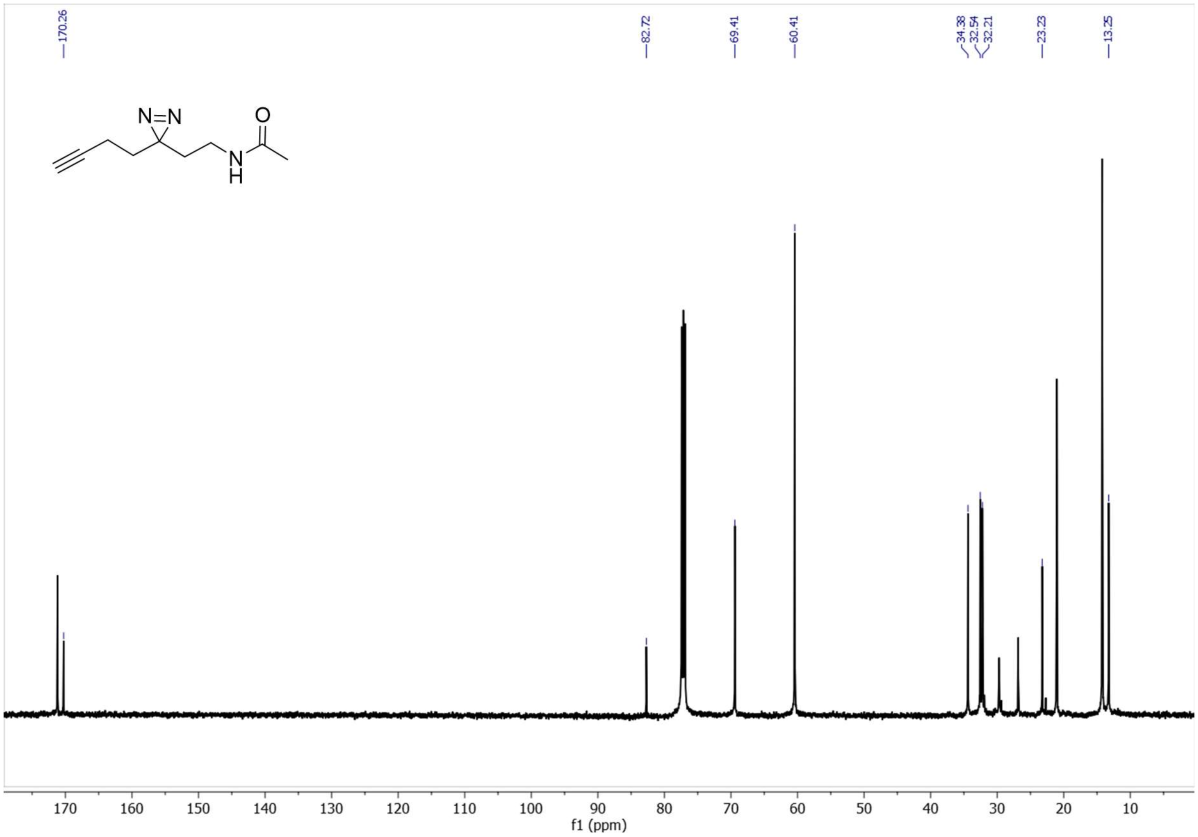
170.26
82.72
69.41
60.41
34.38
32.54
32.21
23.23
13.25
f1 (ppm)

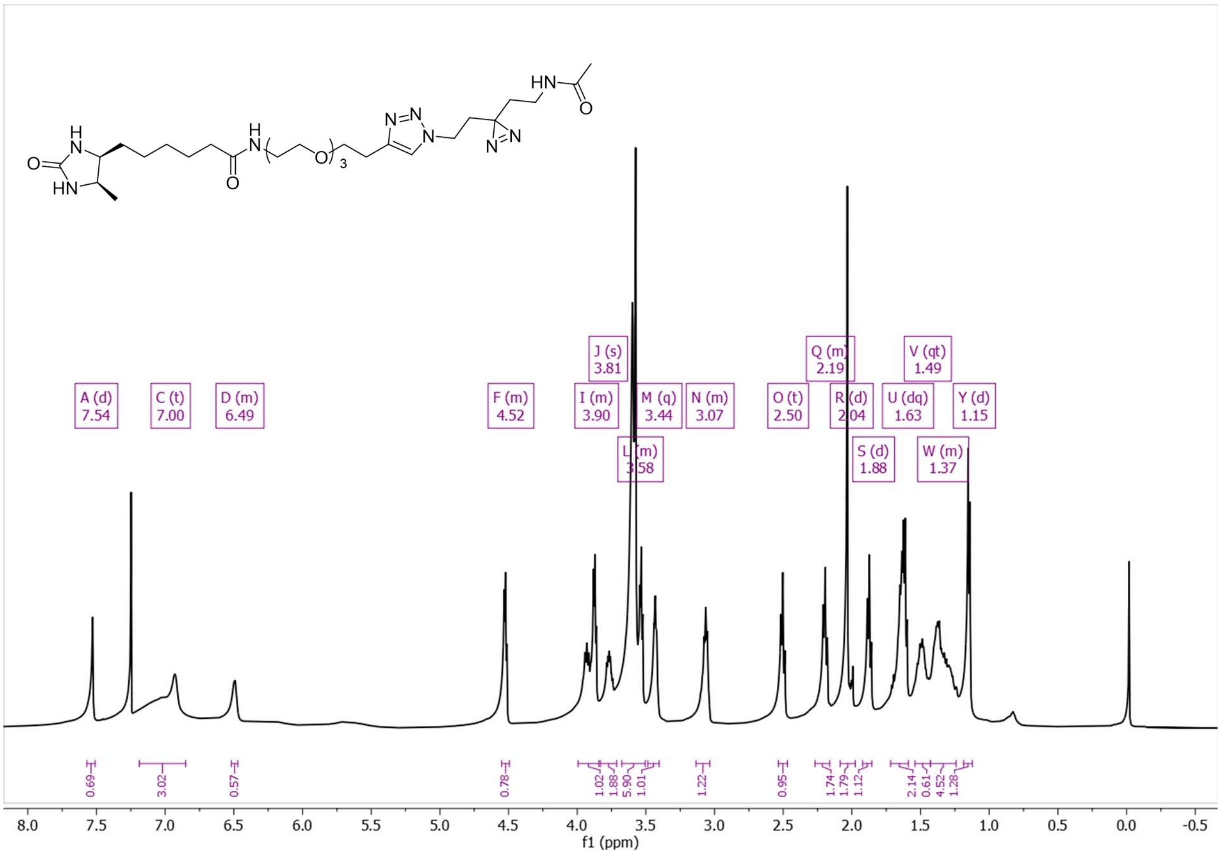
A (d) 7.54
C (t) 7.00
D (m) 6.49
F (m) 4.52
I (m) 3.90
J (s) 3.81
L (m) 3.58
M (q) 3.44
N (m) 3.07
O (t) 2.50
Q (m) 2.19
R (d) 2.04
S (d) 1.88
U (dq) 1.63
V (qt) 1.49
W (m) 1.37
Y (d) 1.15
f1 (ppm)

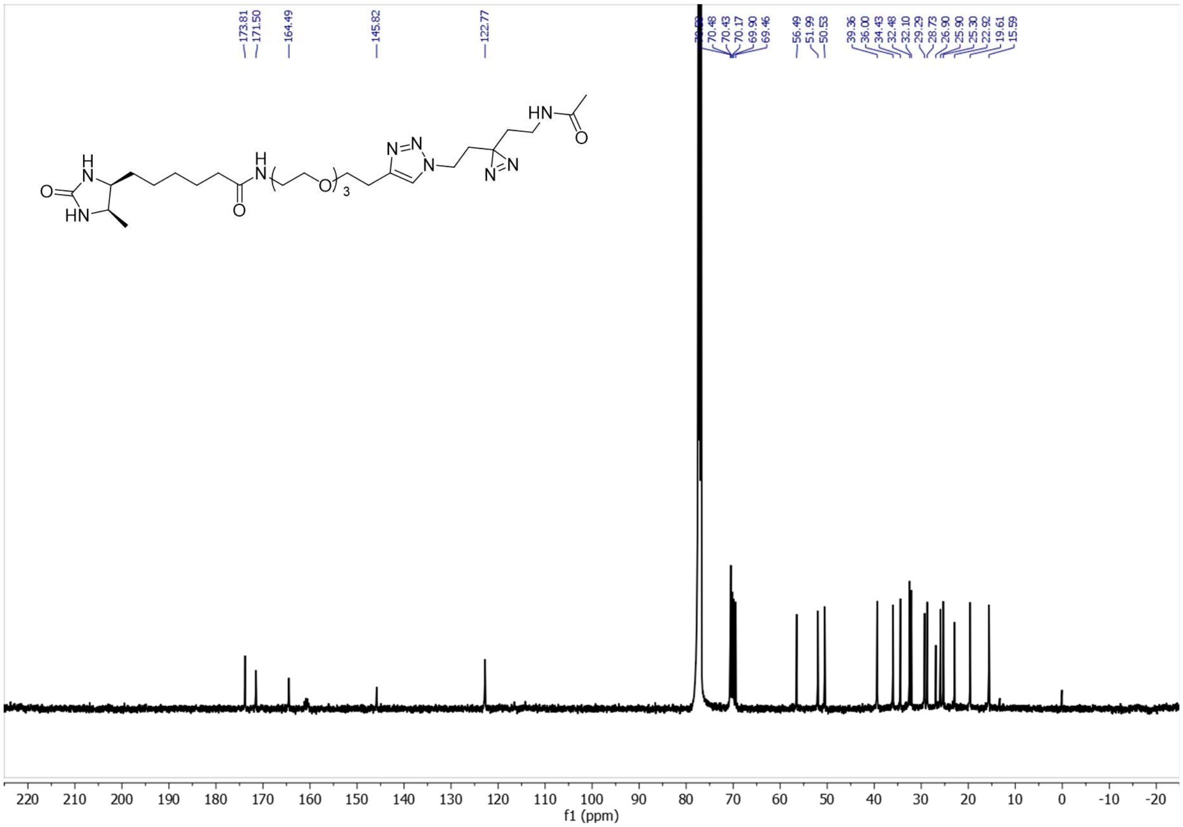
173.81
171.50
164.49
145.82
122.77
70.48
70.43
70.17
69.90
69.46
56.49
51.99
50.53
39.36
36.00
34.43
32.48
32.10
29.29
28.73
26.90
25.90
25.30
22.92
19.61
15.59
HN
O
N=N
N
H
HN
O
N
H
O
3
N=N
220 210 200 190 180 170 160 150 140 130 120 110 100 90 80 70 60 50 40 30 20 10 0 -10 -20
f1 (ppm)

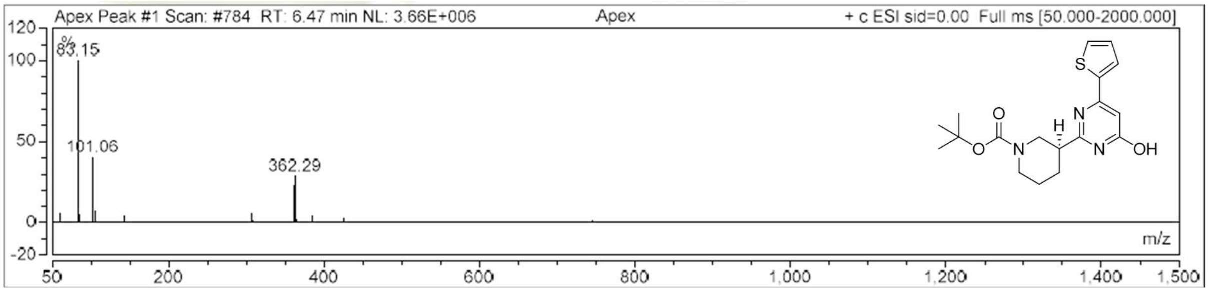
Apex Peak #1 Scan: #784 RT: 6.47 min NL: 3.66E+006
Apex
+ c ESI sid=0.00 Full ms [50.000-2000.000]
%
83.15
101.06
362.29
m/z
OH
S
N
O
H

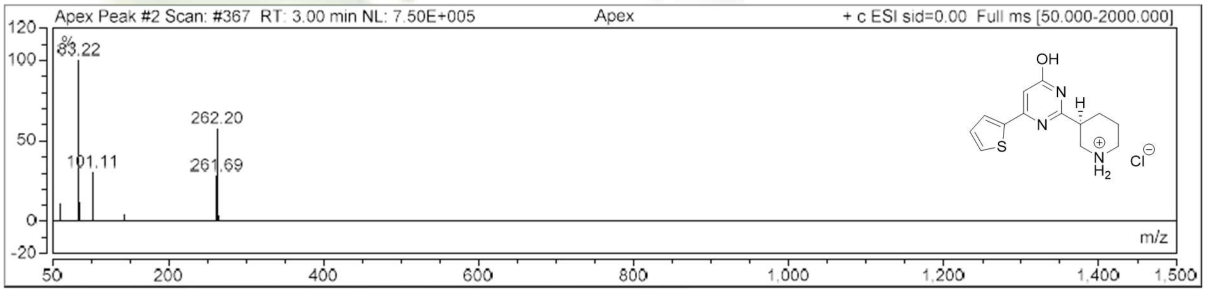
Apex Peak #2 Scan: #367 RT: 3.00 min NL: 7.50E+005
Apex
+ c ESI sid=0.00 Full ms [50.000-2000.000]
%
83.22
262.20
101.11
261.69
m/z
OH
S
Cl

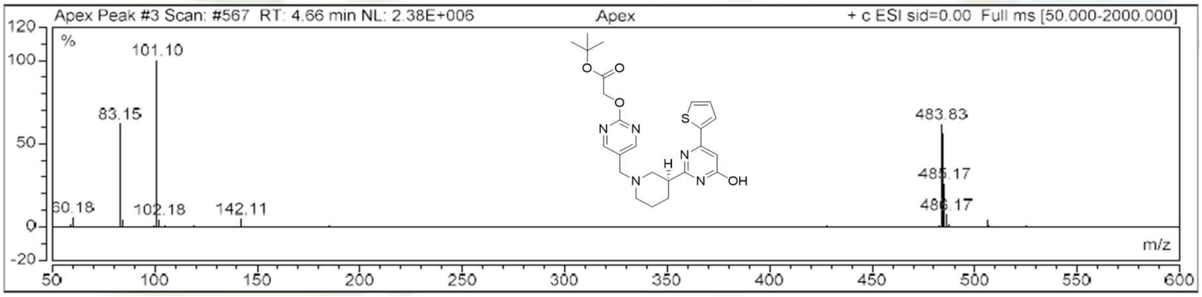
Apex Peak #3 Scan: #567 RT: 4.66 min NL: 2.38E+006
Apex
+ c ESI sid=0.00 Full ms [50.000-2000.000]
%
101.10
83.15
483.83
485.17
486.17
60.18
102.18
142.11
m/z
OH

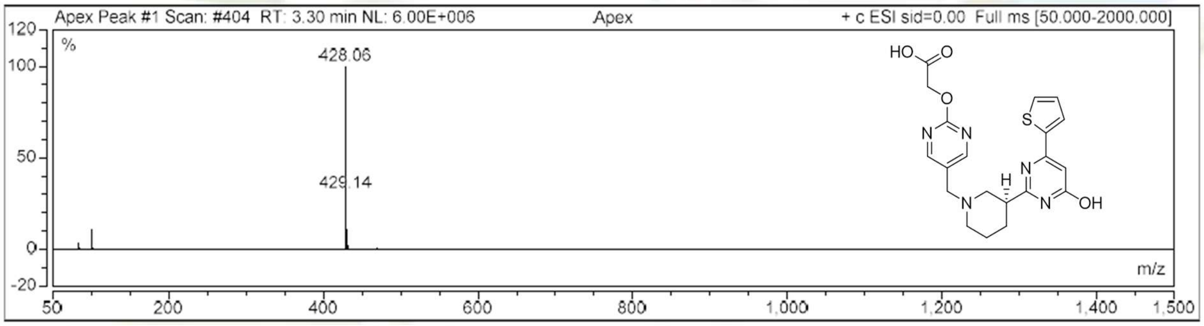
Apex Peak #1 Scan: #404 RT: 3.30 min NL: 6.00E+006
Apex
+ c ESI sid=0.00 Full ms [50.000-2000.000]
%
428.06
429.14
m/z
HO
OH

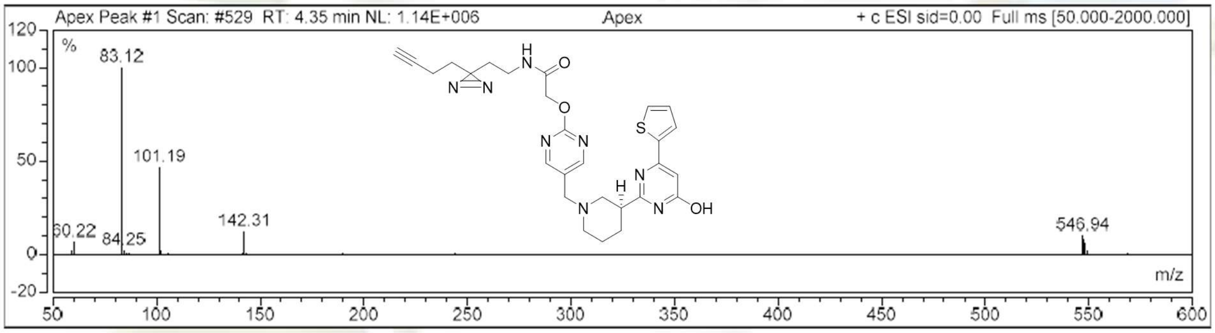
Apex Peak #1 Scan: #529 RT: 4.35 min NL: 1.14E+006
Apex
+ c ESI sid=0.00 Full ms [50.000-2000.000]
%
83.12
101.19
142.31
60.22
84.25
546.94
m/z
OH

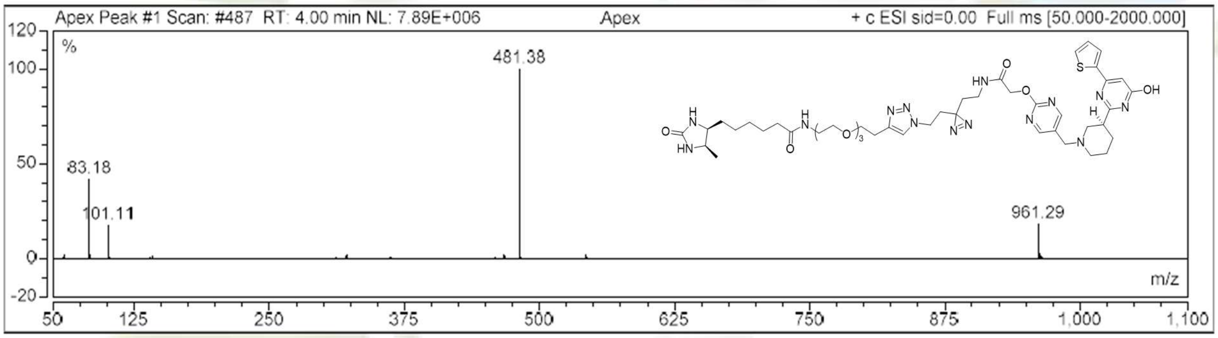

Apex Peak #1 Scan: #487 RT: 4.00 min NL: 7.89E+006
Apex
+ c ESI sid=0.00 Full ms [50.000-2000.000]
%
481.38
83.18
101.11
961.29
m/z

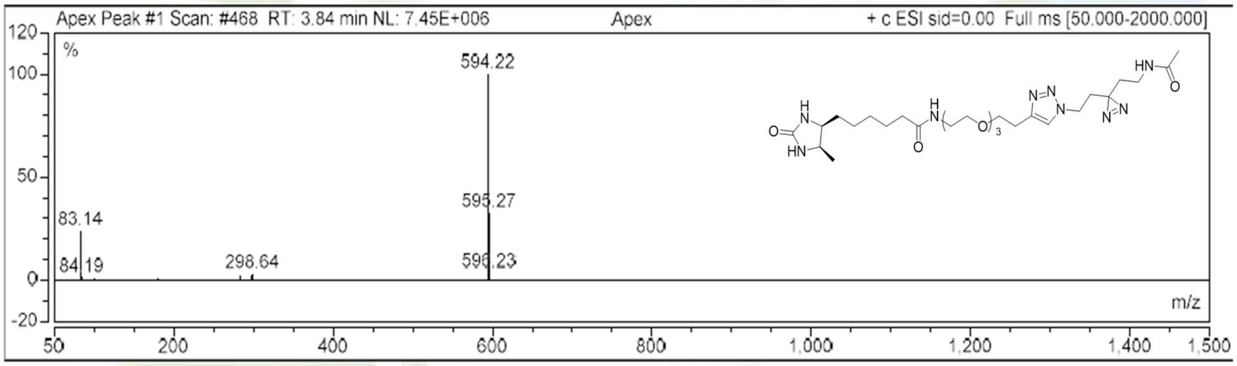

Apex Peak #1 Scan: #468 RT: 3.84 min NL: 7.45E+006
Apex
+ c ESI sid=0.00 Full ms [50.000-2000.000]
%
594.22
595.27
596.23
83.14
84.19
298.64
m/z

References

(1) Motika, S. E.; Ulrich, R. J.; Geddes, E. J.; Lee, H. Y.; Lau, G. W.; Hergenrother, P. J. Gram-Negative Antibiotic Active Through Inhibition of an Essential Riboswitch. *J. Am. Chem. Soc.* **2020**, *142* (24), 10856–10862.
